# IBIS: identify biomarker-based subgroups with a Bayesian enrichment design for targeted combination therapy

**DOI:** 10.1186/s12874-023-01877-w

**Published:** 2023-03-20

**Authors:** Xin Chen, Jingyi Zhang, Liyun Jiang, Fangrong Yan

**Affiliations:** Research Center of Biostatistics and Computational Pharmacy, China Pharmaceutical University, Nanjing, China

**Keywords:** Biomarker, Subgroup identification, Adaptive enrichment design, Combination therapy, Bayesian hierarchical model (BHM), Two-stage design

## Abstract

**Background:**

Combination therapies directed at multiple targets have potentially improved treatment effects for cancer patients. Compared to monotherapy, targeted combination therapy leads to an increasing number of subgroups and complicated biomarker-based efficacy profiles, making it more difficult for efficacy evaluation in clinical trials. Therefore, it is necessary to develop innovative clinical trial designs to explore the efficacy of targeted combination therapy in different subgroups and identify patients who are more likely to benefit from the investigational combination therapy.

**Methods:**

We propose a statistical tool called ‘IBIS’ to Identify BIomarker-based Subgroups and apply it to the enrichment design framework. The IBIS contains three main elements: subgroup division, efficacy evaluation and subgroup identification. We first enumerate all possible subgroup divisions based on biomarker levels. Then, Jensen–Shannon divergence is used to distinguish high-efficacy and low-efficacy subgroups, and Bayesian hierarchical model (BHM) is employed to borrow information within these two subsets for efficacy evaluation. Regarding subgroup identification, a hypothesis testing framework based on Bayes factors is constructed. This framework also plays a key role in go/no-go decisions and enriching specific population. Simulation studies are conducted to evaluate the proposed method.

**Results:**

The accuracy and precision of IBIS could reach a desired level in terms of estimation performance. In regard to subgroup identification and population enrichment, the proposed IBIS has superior and robust characteristics compared with traditional methods. An example of how to obtain design parameters for an adaptive enrichment design under the IBIS framework is also provided.

**Conclusions:**

IBIS has the potential to be a useful tool for biomarker-based subgroup identification and population enrichment in clinical trials of targeted combination therapy.

**Supplementary Information:**

The online version contains supplementary material available at 10.1186/s12874-023-01877-w.

## Background

In recent years, the rapid development of targeted combination therapy has brought novel treatment options for cancer patients. For example, atezolizumab (PD-L1 inhibitor) plus bevacizumab (VEGF inhibitor) could maintain clinically meaningful survival benefits compared with sorafenib in patients with unresectable hepatocellular carcinoma [1]. First-line treatment with nivolumab (PD-1 inhibitor) plus ipilimumab (CTLA4 inhibitor) resulted in a longer duration of overall survival than did chemotherapy in patients with advanced non-small-cell lung cancer [2]. A single-arm, phase Ib-II trial of pembrolizumab (PD-1 inhibitor) plus trastuzumab (HER2 inhibitor) also demonstrated activity and durable clinical benefit in patients with PD-L1-positive, trastuzumab-resistant, advanced, HER2-positive breast cancer [3]. Such combination therapies are directed at multiple therapeutic targets and may improve treatment response, prevent development of resistance, or reduce adverse events. However, the efficacy of targeted combination therapy could be heterogeneous across subgroups and is generally related to the levels of certain predictive biomarkers [4]. Traditional treatment strategy without selecting population is no longer desirable. Compared to monotherapy, subgroup identification is usually more complicated for targeted combination therapy due to the increasing number of subgroups. For example, patients treated with a PD-1 inhibitor are often divided into three subgroups, including PD-L1 less than 1, 1–49%, and 50% or greater, while patients treated with a HER2 inhibitor can be divided into HER2-positive and HER2-negative subgroups. Thus, if these two kinds of therapies are combined for treatment, there are consequently a total of six subgroups. This may result in insufficient sample sizes and slow recruitment for some subgroups, leading to efficacy evaluation challenges in clinical trials. One motivation example of this paper is an ongoing phase 1b, open-label, 2-part, multicenter, non-randomized, multiple-dose study which evaluates DS-8201a in combination with pembrolizumab in participants with advanced/metastatic breast cancer or non-small cell lung cancer (ClinicalTrials.gov Identifier: NCT04042701) [5]. DS-8201a is an anti-HER2 antibody-drug conjugate (ADC) with a novel topoisomerase I inhibitor and pembrolizumab is a PD-1 inhibitor. Therefore, it is highly likely that the efficacy of the drug combination is related to patients’ expression levels of HER2 and PD-L1. The dose expansion part of the study includes breast cancer patients with both HER2-positive and HER2-low-positive, and the inclusion criteria does not limit the expression level of PD-L1. Therefore, although the primary objective of this example is not exactly to identify subgroups based on these two predictive biomarkers, we regard it as a scenario where a data-driven subgroup identification is possible.

Moreover, even if the target populations of both single drugs have been identified through historical studies, we cannot claim that the target population of the combination therapy is simply the intersection of those two populations because combination therapy can potentially enhance efficacy and reduce drug resistance by targeting multiple key pathways in a synergistic or an additive manner [6]. Unlike monotherapy, where there is usually a monotonic relationship between efficacy and biomarker level, the efficacy profiles of combination therapy across subgroups could become more complicated due to the existence of interaction effects. Therefore, it is necessary to develop new innovative clinical trial designs to explore the efficacy of targeted combination therapy in different subgroups and identify patients who are more likely to benefit from the investigational combination therapy.

Another recent change in medical practice is the increasing refinement of biomarker-based subgroup classification, representing a shift from dichotomy to multilevel classification. For example, patients with breast cancer are usually divided into HER2-positive and HER2-negative subgroups in clinical practice. For HER2-positive patients, trastuzumab-based or other HER2-targeted drug regimens are now standards of care [7]. However, recent studies have shown that HER2-low-positive and HER2-zero breast cancers, although generally classified as HER2-negative, are distinct in terms of prognosis and response to treatment [8]. Preclinical studies of DS-8201, an anti-HER2 ADC, indicate that the antitumor activity of the drug is dependent on HER2 expression level rather than on HER2 amplification [9]. An early-phase clinical trial showed that the drug has a certain effect on breast cancer patients with IHC2+ and IHC1+, while IHC1+ is generally classified as HER2-negative [10]. This evolving paradigm of subgroup classification is more consistent with the concept of precision medicine, but it may further increase the number of subgroups, causing challenges with respect to subgroup identification and efficacy evaluation. As the treatment effects may be similar in adjacent biomarker-based subgroups (e.g., IHC2+ and IHC1+), one possible strategy to address this issue is to borrow information across similar subgroups. Therefore, we propose a tool called ‘IBIS’ in this article to detect the potential similarities across subgroups and Identify BIomarker-based Subgroups with higher efficacy.

We also extend IBIS to an adaptive enrichment design framework to increase its applicability. The adaptive enrichment design can adjust the inclusion/exclusion criteria according to a pre-specified plan based on the results of interim analysis, allowing the flexibility to explore the efficacy of investigational drugs for different subgroups. Several adaptive enrichment designs have been proposed, some of which take into account the case of a single dichotomous biomarker [11–21]. In these trial designs, patients regardless of marker status are enrolled at stage I. Then, an interim analysis is performed to decide whether to continue enrolling the entire population or to enroll only biomarker-positive patients. Some other studies considered a more general case of nested subgroups and focused on subgroup selection, assuming multiple pre-specified subgroups with or without a priori ordering [22–26]. However, when it comes to two or more predictive biomarkers, the efficacy of the drug combination may be partially ordered. For example, we can assume the efficacy of PD-1 inhibitors in patients with PD-L1 ≥ 50% will not be lower than that of patients with PD-L1 < 50%; the efficacy of HER2 inhibitors in patients with HER2-positive will not be lower than that of patients with HER2-negative. However, we cannot judge whether a combination of PD-1 inhibitor and HER2 inhibitor is better for patients with PD-L1 ≥ 50% and HER2-negative than for patients with PD-L1 < 50% and HER2-positive. Therefore, those existing designs with or without a priori ordering for subgroups cannot completely meet the requirements of subgroup identification with multiple predictive biomarkers. To the best of our knowledge, there are limited systematic studies on the issues of biomarker-based subgroup identification and population enrichment in terms of targeted combination therapy. There are also few studies considering similarities across adjacent subgroups. Considering the increasing number of subgroups and complicated biomarker-based efficacy profiles, it is of great significance to propose new design methods to identify subgroups and enrich populations for targeted combination therapy.

## Methods

### IBIS design

#### Subgroup division

**Fig. 1:**
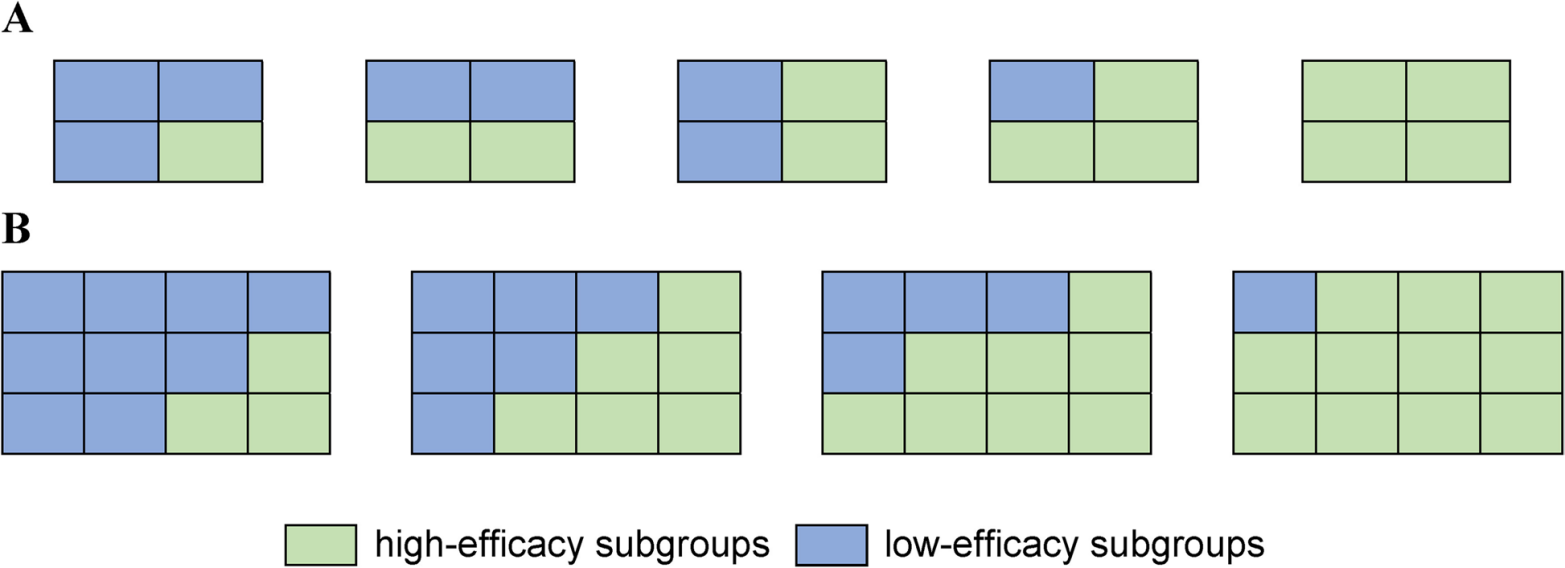
Examples of subgroup divisions. The level of *Biomarker1* increases from top to bottom, and the level of *Biomarker2* increases from left to right. The high-efficacy subgroups and low-efficacy subgroups are represented in green and blue, respectively. **A** Five possible subgroup divisions when *K* = 2 and *J* = 2. **B** Four possible subgroup divisions when *K* = 3 and *J* = 4, and there are 34 divisions in total

Considering a clinical trial whose primary objective is to determine whether a two-agent targeted drug combination (e.g., pembrolizumab plus trastuzumab) is effective for some specific subgroups, we first divide the subgroups into high-efficacy and low-efficacy subsets. This type of division could be an issue with the refinement of biomarker-based subgroup classification. It is assumed that two corresponding biomarkers are incorporated, denoted as *Biomarker1* and *Biomarker2* (e.g., PD-L1 and HER2). The entire population can be divided into *K* ordered subgroups based on *Biomarker1* or *J* ordered subgroups based on *Biomarker2*; thus the total number of subgroups is *K* × *J*. Assuming that the efficacy of both targeted agents increases monotonically with biomarker levels, i.e., marginal monotonicities, a total of *G* high-efficacy subsets Π_*g*_(*g* = 1, …, *G*) can be listed, where the *G*_*th*_ subset Π_*G*_ represents the entire population. Each high-efficacy subset has at least one high-efficacy patient subgroup, i.e., Π_*g*_ ≠ ∅. Taking one of the simplest forms (*K* = 2, *J* = 2) as an example, there are five possible subgroup divisions (*G* = 5, Fig. 1A).

As the number of subgroups increases, the possible situations of subgroup division expand rapidly. For example, when *K* = 3 and *J* = 4, there are 34 possible divisions altogether, four among which are shown in Fig. 1B. A computer algorithm can be used to enumerate all possible divisions satisfying the marginal monotonic assumption. The algorithm is given below.

1. Let $r\left(k,j\right)$ denote the variable indicating the magnitude of efficacy for subgroup$\left(k,j\right)$, where *k *= 1,…, *K* and *j *= 1,…, *J*. To describe the algorithm more conveniently, let $r\left(k,0\right)=r\left(0,j\right)=0$.
2. Use the following loop to sequentially assign values to $r\left(k,j\right)$:
  FOR *k* = 1,…, *K*
  FOR *j* = 1,…, *J*
    DO $$r\left(k,j\right)=\text{runif}\left(\text{max}\left(r\left(k-1,j\right),\;r\left(k,j-1\right)\right),1\right)$$
  where $\text{runif}\left(a,b\right)$ denotes a uniformly distributed random number between *a* and b, and $\text{max}\left(a,b\right)$ denotes the larger of 𝑎 and 𝑏.
3. Sort $r\left(k,j\right)$ to get an ordering.
4. Repeat steps 2 and 3 to get 𝑁 orderings (𝑁 is a large number, such as 10^6^). Eliminate duplicates in these orderings.
5. Partition the orderings to obtain possible situations of the high-efficacy subset.
6. Eliminate duplicates in the obtained divisions without regard to the orderings within subset.

#### Efficacy evaluation

For ease of elucidation, we first consider the scenario of a single-arm trial. Let *Y*_(*k*, *j*)_ denote the efficacy measure outcome for patients in subgroup (*k*, *j*), which follows a one-parameter exponential family distribution, i.e., *Y*_(*k*, *j*)_~*f*(*ψ*_(*k*, *j*)_). For example, if the response rate is the efficacy endpoint, whether a patient in subgroup (*k*, *j*) responds to investigational treatment can be viewed as following a Bernoulli distribution with probability *ψ*_(*k*, *j*)_. As the treatment effects are relatively similar within the high-efficacy and low-efficacy subsets, we transform the original parameter *ψ*_(*k*, *j*)_ into an exchangeable parameter *θ*_(*k*, *j*)_ = *h*(*ψ*_(*k*, *j*)_) in preparation for borrowing information via a hierarchical model. A typical example of the transform function *h*(∙) is the logit function for a binary endpoint. Table 1 shows the situations for some other commonly used endpoints.

**Table 1:** Sampling model, transform function and example of clinical endpoints for different types of one-parameter exponential families

*f*(*ψ*_(*k*, *j*)_)	*h*(*ψ*_(*k*, *j*)_)	Example of clinical endpoint
*Normal*(*ψ*_(*k*, *j*)_, *σ*^2^ )	*ψ*_(*k*, *j*)_	Percentage change in tumor size
*Bernoulli*(*ψ*_(*k*, *j*)_)	logit(*ψ*_(*k*, *j*)_)	Tumor response rate
*Exponential*(*ψ*_(*k*, *j*)_)	log(*ψ*_(*k*, *j*)_)	Progression free survival
*Poisson*(*ψ*_(*k*, *j*)_)	log(*ψ*_(*k*, *j*)_)	Number of tumor-involved nodes

*f*(∙), sampling model; *h*(∙), transform function. The nuisance parameter *σ*^2^ is assumed to be known for a normal distribution

One simple way to model the efficacy outcome is applying Bayesian hierarchical models to borrow information across all subgroups. However, when the heterogeneity across subgroups is large, using this strategy may lead to substantial bias. Therefore, it is more preferable to classify subgroups into two subsets based on accumulated data and then borrow information within each subset. Let Π_∁*g*_ (low-efficacy subset) denote the complementary set for subset Π_*g*_ (high-efficacy subset). A Bayesian hierarchical model is constructed as follows to borrow information within the high-efficacy and low-efficacy subsets:

$${\theta}_{\left(k,j\right)}\sim N\left({\theta}_g,{\sigma}_g^2\right),\textrm{if}\ \left(k,j\right)\in {\Pi}_g,$$

$${\theta}_{\left(k,j\right)}\sim N\left({\theta}_{\complement g},{\sigma}_{\complement g}^2\right),\textrm{if}\ \left(k,j\right)\in {\Pi}_{\complement g},$$

where *θ*_*g*_ and *θ*_∁*g*_ denote the average treatment effects for high-efficacy and low-efficacy subsets, respectively. Shrinkage parameters ${\sigma}_g^2$ and ${\sigma}_{\complement g}^2$ are the intersubgroup variances of treatment effects within these two subsets, controlling the degree of information borrowing. They do not need to be specified in advance and can be data-driven. If treatment effect estimates across subgroups within a subset are similar, then the posteriors of the intersubgroup variance will be smaller, inducing a strong borrowing. If treatment effect estimates across subgroups within a subset are very different, then less borrowing will occur. Normal distributions with large variances are usually taken as the priors for *θ*_*g*_ and *θ*_∁*g*_. In terms of the priors for ${\sigma}_g^2$ and ${\sigma}_{\complement g}^2$, an inverse-gamma distribution *IG*(*a*, *b*) can be adopted. Small values of *a* and *b* are set such that the priors are vague. We constrain *θ*_*g*_ > *θ*_∁*g*_ to avoid the potential computational issue of label switching when using the Gibbs sampler to sample posteriors.

To distinguish high-efficacy and low-efficacy subgroups, we use Jensen–Shannon divergence [27] to measure the distance between the two posterior distributions of the average treatment effect *θ*_*g*_ and *θ*_∁*g*_, which is also a measure of the similarity between high-efficacy subset and low-efficacy subset:

$$\textrm{JSD}\left({\theta}_g\Big\Vert {\theta}_{\complement g}\right)=\frac{1}{2}{D}_{KL}\left({\theta}_g\Big\Vert \overset{\sim }{\theta}\right)+\frac{1}{2}{D}_{KL}\left({\theta}_{\complement g}\Big\Vert \overset{\sim }{\theta}\right)$$

where $\overset{\sim }{\theta }=\frac{1}{2}\left({\theta}_g+{\theta}_{\complement g}\right)$. *D*_*KL*_(*A*‖*B*) denotes the Kullback–Leibler divergence between *A* and *B*, which is defined as follows when *A* and *B* are both continuous variables:

$${D}_{KL}\left(A\Big\Vert B\right)=\int a(x)\log \left(\frac{a(x)}{b(x)}\right) dx,$$

where *a*(*x*) and *b*(*x*) are the probability densities of *A* and *B*, respectively. After calculating the Jensen–Shannon divergences for all subgroup divisions, the optimal division result is defined as the division that maximizes the Jensen–Shannon divergence between *θ*_*g*_ and *θ*_∁*g*_, because that is when Π_*g*_ and Π_∁*g*_ are most dissimilar. Let *C*_*H*_ denote the high-efficacy subset in the optimal division:

$${C}_H=\underset{\Pi_g}{\textrm{argmax}}\left\{\textrm{JSD}\left({\theta}_g\Big\Vert {\theta}_{\complement g}\right),g=1,\dots, G-1\right\}.$$

Based on this optimal division, the posterior distribution of the treatment effect for each subgroup *θ*_(*k*, *j*)_ can be obtained by applying the aforementioned Bayesian hierarchical model. We use the Jensen–Shannon divergence here because it is based on the well-known Kullback–Leibler divergence, and it has the property of symmetry. Some other measures of distance between distributions, such as the Hellinger distance [28], may also be applicable.

The model introduced above can be easily extended to randomized controlled trials (RCTs). The treatment effects of the investigational drug and the control intervention for subgroup (*k*, *j*) are ${\theta}_{\left(k,j\right)}^T$ and ${\theta}_{\left(k,j\right)}^C$, respectively. Therefore, the effect size of interest ${\theta}_{\left(k,j\right)}={\theta}_{\left(k,j\right)}^T-{\theta}_{\left(k,j\right)}^C$, and the above statistical model can still be applied.

#### Subgroup identification

The objective of subgroup identification is to find a collection of subgroups with clinically meaningful treatment effects. Suppose the hypothesis is as follows:

$${H}_{0\left(k,j\right)}:{\theta}_{\left(k,j\right)}\le {\theta}_0,{H}_{1\left(k,j\right)}:{\theta}_{\left(k,j\right)}>{\theta}_0\ \textrm{for}\ k=1,\dots, K,j=1,\dots, J.$$

In single-arm trials, *θ*_0_ represents the minimum acceptable treatment effect, which is usually equal to the efficacy of the existing standard of care. In randomized controlled trials, *θ*_0_ is the superiority margin and is usually taken as 0.

Bayes factors are used to test the hypothesis in IBIS. If the Bayes factor *BF*_(*k*, *j*)_ corresponding to subgroup (*k*, *j*) is sufficiently large, i.e.,

$${BF}_{\left(k,j\right)}=\frac{\Pr \left({H}_{1\left(k,j\right)}|D\right)/\Pr \left({H}_{0\left(k,j\right)}|D\right)}{\Pr \left({H}_{1\left(k,j\right)}\right)/\Pr \left({H}_{0\left(k,j\right)}\right)}>{BF}_{E\left(k,j\right)},$$

the investigational combination therapy is considered to be effective for subgroup (*k*, *j*), where *D* denotes the accumulated trial data and *BF*_*E*(*k*, *j*)_ is a pre-specified threshold for (*k*, *j*). Under the Bayesian paradigm, we assign each of the hypotheses a prior probability of being true, as denoted by Pr(*H*_0(*k*, *j*)_) and Pr(*H*_1(*k*, *j*)_). Correspondingly, the posterior probabilities are denoted as Pr(*H*_0(*k*, *j*)_| *D*) and Pr(*H*_1(*k*, *j*)_| *D*). To satisfy the assumption that efficacy increases monotonically with biomarker levels, once the investigational therapy is considered effective for subgroup $\left(\overset{\sim }{k},\overset{\sim }{j}\right)$, it is also deemed effective for subgroups in subset $C:\left\{k\ge \overset{\sim }{k},j\ge \overset{\sim }{j}\right\}$.

We do not directly judge whether the investigational therapy is effective for *C*_*H*_ (i.e., making inference on *θ*_*g*_ at the subset level) because this is a kind of ‘statistical’ subgroup division, not a ‘clinical’ one. In addition, such a subset can always be identified as mentioned before. If the therapy is ineffective for all subgroups, making such judgments will inevitably lead to type I errors; if the therapy is effective for all subgroups, it will exclude some subgroups and result in type II errors. The main purpose for subgroup division in IBIS is to reduce the bias of estimation generated by BHM when there is heterogeneity across subgroups rather than directly inferring and making decisions based on the division results.

The reason why we use the Bayes factors rather than directly using the posterior probability Pr(*H*_1(*k*, *j*)_| *D*) or the posterior odds Pr(*H*_1(*k*, *j*)_| *D*)/ Pr(*H*_0(*k*, *j*)_| *D*) to make decisions is mainly that when the intervals corresponding to the null and alternative hypotheses are not the same length, such as a point null hypothesis, the posterior probability or the posterior odds may not reflect what we really want to quantify. Suppose that there is a null hypothesis *H*_0(*k*, *j*)_ : *θ*_(*k*, *j*)_ = *θ*_0_ and an alternative hypothesis *H*_1(*k*, *j*)_ : *θ*_(*k*, *j*)_ ≠ *θ*_0_. For posterior odds Pr(*H*_1(*k*, *j*)_| *D*)/ Pr(*H*_0(*k*, *j*)_| *D*), it is always going to get an infinity result which cannot used for decision-making. However, in terms of Bayes factor, the Pr(*H*_1_| *D*)/ Pr(*H*_1_) is approximately equal to 1. So we can get the Bayes factor by calculating Pr(*H*_0_)/ Pr(*H*_0_| *D*), i.e., the ratio of the prior density and the posterior density. Therefore, Bayes factors can increase the flexibility in formulating relevant hypotheses. On the other hand, although the the posterior probability or the posterior odds value can represent the strength of evidence in favour of the alternative hypothesis, the Bayes factor is a more commonly used Bayesian solution to the hypothesis testing problems.

#### Extension to adaptive enrichment design

Along the way of subgroup identification, IBIS can be extended to the following multi-stage adaptive enrichment design. Suppose there are a total of *I* analyses, including *I* − 1 interim analyses and one final analysis. Patients with any biomarker status can be enrolled in the initial stage of the trial. In the *i*_*th*_(*i* = 1, …, *I* − 1) interim analysis, a go/no-go decision is made based on the Bayes factors, specifically as follows:

1. If ${BF}_{\left(k,j\right)}>{BF}_{E\left(k,j\right)}^{(i)}$, the investigational therapy is considered to be effective for subgroup (*k*, *j*);
2. If ${BF}_{\left(k,j\right)}\le {BF}_{P\left(k,j\right)}^{(i)}$, the investigational therapy is considered to be ineffective for subgroup (*k*, *j*);
3. If *BF*_(*k*, *j*)_ is between these two thresholds, the investigational therapy is considered promising for subgroup (*k*, *j*).

**Fig. 2:**
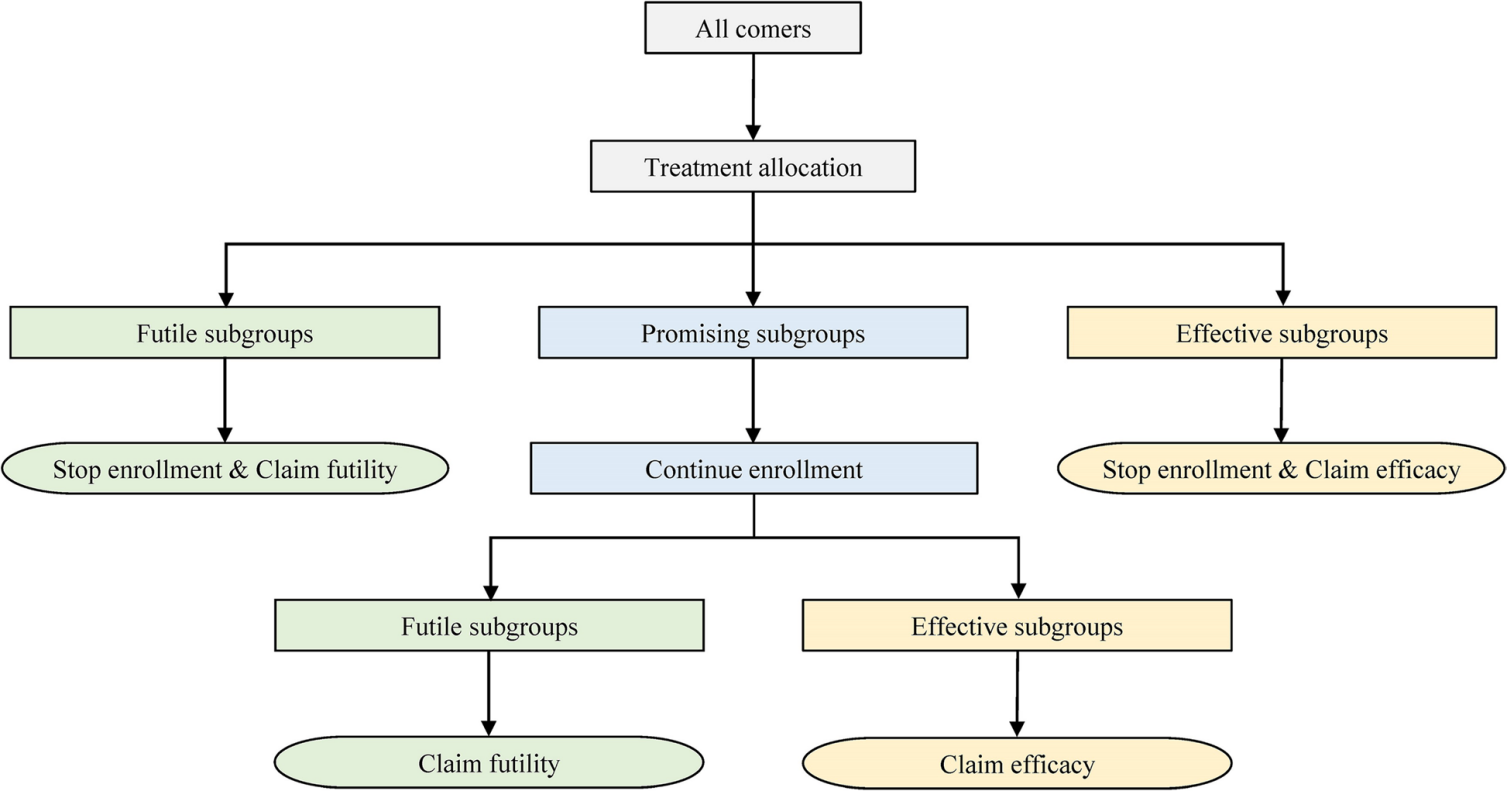
Schema of a two-stage adaptive enrichment design based on IBIS

Thresholds ${BF}_{E\left(k,j\right)}^{(i)}$ and ${BF}_{P\left(k,j\right)}^{(i)}$ are important design parameters that need to be calibrated carefully, and the calibration strategy will be covered later. After the interim analysis, only promising subgroups will be enrolled in the next stage. The enrollment of the other two kinds of subgroups will be stopped early for efficacy or futility. The whole trial will be stopped early if there are no promising subgroups. Note that we can define ${BF}_{E\left(k,j\right)}^{(i)}=\infty \left(i=1,\dots, I-1\right)$ and thus prevent early stopping for efficacy. At the final analysis, if ${BF}_{\left(k,j\right)}>{BF}_{E\left(k,j\right)}^{(I)}$, the investigational therapy is considered to be effective for subgroup (*k*, *j*); otherwise, the investigational therapy is considered to be ineffective for subgroup (*k*, *j*). It is logistically and operationally intractable to change enrollment criteria too often in one clinical trial and a two-stage design is often recommended for the adaptive enrichment design. Figure 2 shows the schema of a two-stage adaptive enrichment design based on IBIS.

We also considered the monotonic relationship between drug efficacy and biomarker levels here. Specifically, once the investigational therapy is considered promising for subgroup $\left(\overset{\sim }{k},\overset{\sim }{j}\right)$, it is also deemed promising for subgroups which were initially judged ineffective in subset $C:\left\{k\ge \overset{\sim }{k},j\ge \overset{\sim }{j}\right\}$; once the investigational therapy is considered effective for subgroup $\left(\overset{\sim }{k},\overset{\sim }{j}\right)$, it is also deemed effective for subgroups which were initially judged ineffective or promising in subset $C:\left\{k\ge \overset{\sim }{k},j\ge \overset{\sim }{j}\right\}$.

Since the proposed adaptive enrichment design incorporates the strategy of early stopping for futility and efficacy, it may face challenges including type I error inflation and reduced statistical power. Therefore, it is critical to determine reasonable design parameters in the planning stage. Usually, two key metrics, family-wise error rate (FWER) and conjunctive power [29], are the main concern; these metrics are defined as follows:

$$FWER=\Pr \left( Reject\ at\ least\ one\ {H}_{0\left(k,j\right)}|{H}_{0\left(k,j\right)}\ is\ true\right)$$

$$Conjunctive\ Power=\Pr \left( Reject\ all\ {H}_{0\left(k,j\right)}|{H}_{1\left(k,j\right)}\ is\ true\right)$$

The general strategy of calibrating design parameters is to make the design achieve satisfactory FWER, conjunctive power and expected sample size in several typical scenarios by simulation.

### Simulation study

#### Evaluation of the estimation performance

We conduct computer simulations to evaluate the estimation performance of IBIS. Consider a one-stage trial where the primary efficacy endpoint is the ratio of tumor size at 1 month after treatment to that at baseline. After transformation to the log scale, this ratio is assumed to be a normally distributed continuous variable. The smaller the log of the ratio, the greater the benefit to patients. To be consistent with the hypothesis testing framework described before, we add a minus sign to the transformed endpoint. Using statistical notation, if *Y*_(*k*, *j*)_ denotes the ratio of tumor size for subgroup (*k*, *j*), then $-\log \left({Y}_{\left(k,j\right)}\right)\sim N\left({\theta}_{\left(k,j\right)},{\sigma}_{\left(k,j\right)}^2\right)$. There are three and four levels for *Biomarker1* and *Biomarker2*, respectively, so the entire population can be divided into 12 subgroups. The minimum acceptable treatment effect *θ*_0_ = 0 and a clinically meaningful treatment effect is equal to 1. A total of eight scenarios are incorporated in the simulation, including the global null (scenario 1), global alternative (scenario 2), good nugget (scenario 3), bad nugget (scenario 4), mostly null (scenario 5), mostly alternative (scenario 6), half alternative (scenario 7) and linear (scenario 8) scenarios. The detailed treatment effect *θ*_(*k*, *j*)_ for each subgroup can be found in Table 2. It is assumed that all ${\sigma}_{\left(k,j\right)}^2$ equal 1, and the sample size of each subgroup is 10. The priors are set as follows: *θ*_*g*_~*N*(1, 10^3^), *θ*_∁*g*_~*N*(0, 10^3^), ${\sigma}_g^2\sim IG\left({10}^{-3},{10}^{-3}\right)$ and ${\sigma}_{\complement g}^2\sim IG\left({10}^{-3},{10}^{-3}\right)$.

**Table 2:** Eight scenarios of treatment effects for patients with different biomarker levels

	*Biomarker2*							
Level	1	2	3	4	1	2	3	4
*Biomarker1*	*Scenario 1*				*Scenario 2*			
1	0	0	0	0	**1**	**1**	**1**	**1**
2	0	0	0	0	**1**	**1**	**1**	**1**
3	0	0	0	0	**1**	**1**	**1**	**1**
	*Scenario 3*				*Scenario 4*			
1	0	0	0	0	0	**1**	**1**	**1**
2	0	0	0	0	**1**	**1**	**1**	**1**
3	0	0	0	**1**	**1**	**1**	**1**	**1**
	*Scenario 5*				*Scenario 6*			
1	0	0	0	0	0	0	**1**	**1**
2	0	0	**1**	**1**	0	0	**1**	**1**
3	0	0	**1**	**1**	**1**	**1**	**1**	**1**
	*Scenario 7*				*Scenario 8*			
1	0	0	0	**1**	0	0.25	0.5	**1**
2	0	0	**1**	**1**	0.25	0.5	**1**	**1.25**
3	0	**1**	**1**	**1**	0.5	**1**	**1.25**	**1.5**

Subgroups with clinically meaningful treatment effect are shown in boldface

The metrics for evaluating the estimation performance include the mean squared error (MSE), bias and average width of the 95% equal-tailed credible interval for the posterior distribution of *θ*_(*k*, *j*)_. The MSE is defined as the average squared difference between the estimated values and the actual value of *θ*_(*k*, *j*)_. The bias is defined as the expected difference between the estimated values and the actual value of *θ*_(*k*, *j*)_. For simplification, we omit the subscript (*k*, *j*) when it does not cause ambiguity:

$$MSE=E\left\{{\left(\hat{\theta}-{\theta}^{\ast}\right)}^2\right\}\approx \frac{1}{n_{sim}}\sum_{i=1}^{n_{sim}}{\left({\hat{\theta}}_i-{\theta}^{\ast}\right)}^2,$$

$$Bias=E\left(\hat{\theta}-{\theta}^{\ast}\right)\approx \frac{1}{n_{sim}}\sum_{i=1}^{n_{sim}}\left({\hat{\theta}}_i-{\theta}^{\ast}\right),$$

where $\hat{\theta}$ and *θ*^∗^ denote the estimated value and the actual value of the treatment effect, respectively. ${\hat{\theta}}_i$ denotes the estimated value of the treatment effect for the *i*_*th*_ simulated trial, where we use the posterior mean as the estimate. The total number of simulated trials, denoted as *n*_*sim*_, is 10,000 here. The average width of the 95% equal-tailed credible interval for the posterior distribution is defined as follows:

$$Width=E\left\{l\left(0.975,\theta \right)-l\left(0.025,\theta \right)\right\}\approx \frac{1}{n_{sim}}\sum_{i=1}^{n_{sim}}\left\{l\left(0.975,{\theta}_i\right)-l\left(0.025,{\theta}_i\right)\right\}$$

where *l*(*q*, *θ*) = {*l* : Pr(*θ* ≤ *l*) = *q*}, which denotes a quantile function. *θ*_*i*_ represents the posterior distribution of the treatment effect for the *i*_*th*_ simulated trial.

Estimation methods used for comparison include independent analysis and BHM. In the independent analysis, the parameter estimate is the sample mean $\overline{Y}$. Analogous to the 95% credible interval described above, the precision of the estimate is measured by the width of the 95% confidence interval under the frequentist statistics, i.e., $\overline{Y}\pm {t}_{1-\alpha /2}\left(n-1\right)\times \frac{s}{\sqrt{n}}$, where *t*_1 − *α*/2_(*n* − 1) is the 1 − *α*/2 quantile of the *t* distribution with degrees of freedom equal to *n* − 1 (*α* = 0.05 here). The sample standard deviation $s=\sqrt{\frac{1}{n-1}\sum_{i=1}^n{\left({Y}_i-\overline{Y}\right)}^2}$ and *n* is the sample size of the subgroup under evaluation. In BHM, all *θ*_(*k*, *j*)_ are treated as exchangeable, i.e., borrowing information is conducted across all subgroups. We force ${\theta}_{\left(k,j\right)}\sim N\left(\overset{\sim }{\theta },{\overset{\sim }{\sigma}}^2\right)$ with the priors of $\overset{\sim }{\theta }$ and ${\overset{\sim }{\sigma}}^2$ equal to *N*(0, 10^3^) and *IG*(10^−3^, 10^−3^), respectively.

#### Evaluation of the operating characteristics

The operating characteristics of IBIS on subgroup identification are also evaluated by computer simulation. The simulation settings are the same as those of the above evaluation of the estimation performance. The evaluation metrics include FWER and conjunctive power. The methods used for comparison include BHM, independent analysis and another frequentist subgroup identification method, denoted as ‘Freq’ here. Suppose there is only one subgroup, then with a null hypothesis *H*_0_ : *θ* ≤ 0, an expected treatment effect to be 1 and a standard deviation to be 1, a study with 10 participants has approximately 90% power to reject the null hypothesis using t-test at the 5% significance level. Therefore, it can be expected that if the FWER is controlled when there are 12 subgroups (each with 10 participants), then the conjunctive power will decrease a lot, i.e., resulting in poor performance on subgroup identification. In this case, we presume that using IBIS to borrow information across subgroups may improve the accuracy of subgroup identification.

The decision-making process of BHM is the same as that of IBIS. With the vague prior distributions we set for parameters in IBIS and BHM method, there is no preference for the null hypothesis or the alternative hypothesis with regard to any one subgroup, i.e., Pr(*H*_0(*k*, *j*)_) = Pr(*H*_1(*k*, *j*)_) = 0.5, in the simulation of BHM and IBIS. The decision-making process of independent analysis is nearly the same as that of the IBIS, and the only change is to replace the Bayes factor with a decision-making based on a t-test. If the following inequation

$${t}_{\left(k,j\right)}=\frac{\sqrt{n_{\left(k,j\right)}}\left({\overline{Y}}_{\left(k,j\right)}-{\theta}_0\right)}{s_{\left(k,j\right)}}>{t}_{E\left(k,j\right)}$$

is satisfied, then the investigational combination therapy is considered effective for subgroup (*k*, *j*). *n*_(*k*, *j*)_, ${\overline{Y}}_{\left(k,j\right)}$ and *s*_(*k*, *j*)_ denote the sample size, sample mean and sample standard deviation of subgroup (*k*, *j*), respectively.

In the Freq method, decision-making is based on Lai et al. [24], who first divide the subgroups into two subsets and then make inferences for the subsets separately. Different from using Jensen–Shannon divergence, the high-efficacy subset is determined by selecting the largest t-test statistic, which is defined as follows:

$${C}_H=\underset{\Pi_g}{\textrm{argmax}}\left\{{t}_{\Pi_g},g=1,\dots, G-1\right\}.$$

where ${t}_{\Pi_g}$ is the t-test statistic for subset Π_*g*_. Then, t tests are performed for *C*_*H*_ and its complementary set, respectively. If the test statistic is greater than a pre-specified threshold, the investigational combination therapy is judged to be effective for that subset; otherwise, it will be considered ineffective.

Let the test thresholds for each subgroup in the same method be equal to facilitate evaluation, although adjustments can be made in practice considering the anticipated efficacy and prevalence of each subgroup. In the Freq method, the test thresholds for high-efficacy and low-efficacy subsets are equal. To make the four methods comparable, we calibrate the thresholds to enable their FWER in the global null scenario (scenario 1) to be controlled within 0.1 or 0.05. Specifically, we performed a series of simulation studies for each method under the null scenario over a grid of the thresholds (i.e., the thresholds of Bayes factors for IBIS and BHM; the thresholds of t-test statistics for independent analysis and the Freq method). Then, for each method, the minimum threshold with simulated FWER less than or equal to 0.1 (or 0.05) would be determined as the design parameter.

Regarding the adaptive enrichment design, the parameter calibration is much more complicated. A general strategy is to make the design achieve an acceptable FWER, conjunctive power and expected sample size in some typical scenarios by simulation with a limited maximum sample size. This may be computationally expensive, which is the sacrifice for adaptability and flexibility.

Consider a two-stage trial with a sample size of five for each subgroup in the first stage. If the investigational therapy is promising for a subgroup, five more patients will be enrolled in the second stage. To preliminarily conduct the parameter calibration, let the decision thresholds for each subgroup be the same, i.e., ${BF}_{E\left(k,j\right)}^{(1)}={BF}_{E\left(k,j\right)}^{(2)}={BF}_E$ and ${BF}_{P\left(k,j\right)}^{(1)}={BF}_P$. Specifying loose decision thresholds may increase power for high-efficacy subgroups, but may also inflate type I errors for low-efficacy subgroups. Therefore, we define a decision score function to comprehensively measure FWER, conjunctive power and expected sample size:

$$Score=Power-\beta_1\cdot FWER+\beta_2\cdot\left(1-\frac{EN}{N_{max}}\right)$$

where *EN* denotes the expected sample size and *N*_*max*_ denotes the maximum sample size, which is equal to 120 here. The above function shows that the loss of increasing one unit FWER can offset the benefit of increasing *β*_1_ unit conjunctive power; the loss of increasing one unit *EN* can offset the benefit of increasing *β*_2_/*N*_*max*_ unit conjunctive power. For example, setting *β*_1_ = 1 and *β*_2_ = 0.5 means that a 1% increase of the FWER is enough to offset a 1% increase of power. At the same time, an increase of one expected sample size could offset either a 0.4% (i.e., *β*_2_/*N*_*max*_) increase of power or a 0.4% decrease of the FWER. Such a decision score function can be interpreted as the tradeoff between FWER, conjunctive power and expected sample size. If a large value is set for *β*_1_, the design will favor a stricter control for FWER. The larger the *β*_2_, the more inclined we are to control the cost of the current trial by reducing the expected sample size. How to choose *β*_1_ and *β*_2_ is a key and difficult problem, and is mainly determined by the potential losses caused by type I and type II errors. The type I error results in the loss of future clinical research with ineffective investigational therapeutics, while the type II error indicates the unavailability of effective treatments for some patients and the loss of marketing revenues. How do sponsors view these potential losses and the cost of the current trial will play a decisive role in determining *β*_1_ and *β*_2_. With *β*_1_ = 1 and *β*_2_ = 0.5, scenario 8 is taken as an example here to explain how to obtain design parameters for such an adaptive enrichment design.

## Results

### Accuracy and precision

**Fig. 3:**
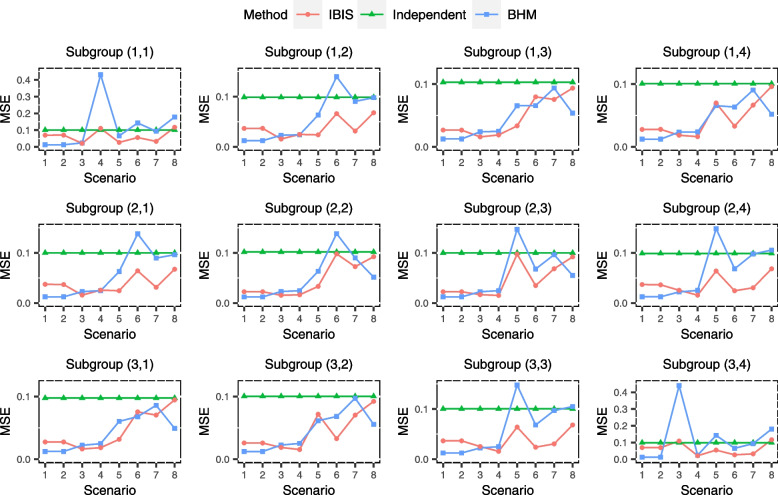
Simulated mean squared error (MSE) of treatment effect estimates for 12 subgroups under eight scenarios using ‘IBIS’, ‘Independent Analysis’ and ‘BHM’

**Fig. 4:**
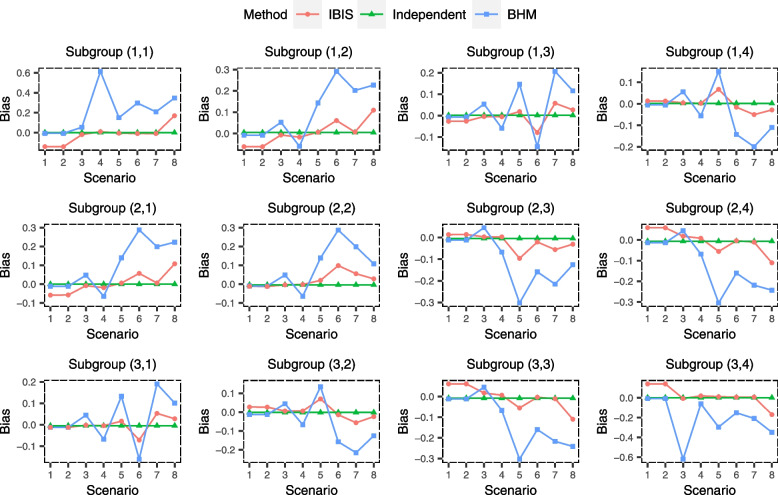
Simulated bias of treatment effect estimates for 12 subgroups under eight scenarios using ‘IBIS’, ‘Independent Analysis’ and ‘BHM’

**Fig. 5:**
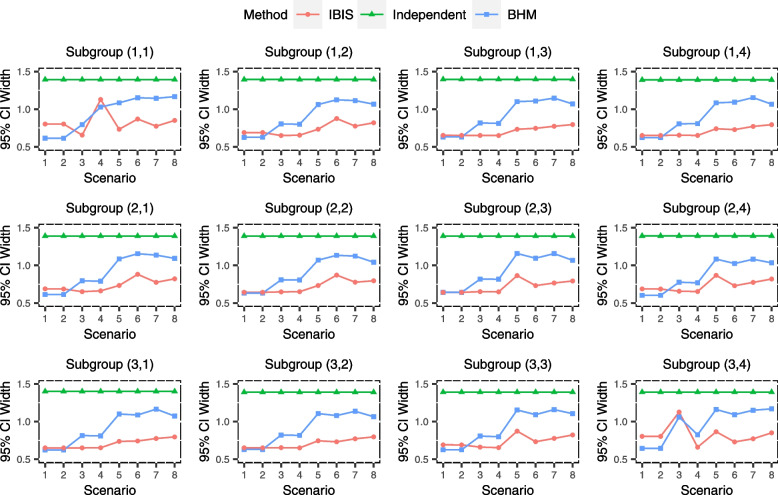
Simulated average width of 95% CI of treatment effect estimates for 12 subgroups under eight scenarios using ‘IBIS’, ‘Independent Analysis’ and ‘BHM’. CI means credible interval in ‘IBIS’ and ‘BHM’ method, while it means confidence interval in ‘Independent Analysis’

The simulation results of the estimation performance are shown in Figs. 3, 4 and 5 and could reflect the accuracy and precision of IBIS. It can be seen that IBIS has the lowest MSE overall, with only few scenarios where the MSEs for few subgroups are slightly higher than that of independent analysis (Fig. 3). Specifically, in subgroup (3,4) of scenario 3 (the good nugget), the MSE of IBIS is relatively high. This is because the treatment effect for this subgroup is so different from other subgroups, and IBIS tends to analyze it alone or just combine it with few adjacent subgroups, resulting in a relatively large variance of the estimate, which can be demonstrated by its wide 95% CI as well (Fig. 5). In this nugget scenario, BHM inevitably produces a large estimation bias (Fig. 4), which in turn leads to a much higher MSE. Similar results also arise in subgroup (1,1) of scenario 4. The estimate of the independent analysis for each subgroup is unbiased (Fig. 4), so the MSE is consistent with the variance of the sample mean, i.e., standard error, which is a constant of 0.1 (Fig. 3). The MSE of BHM is the smallest in homogeneous scenarios (scenarios 1 and 2), but BHM is not robust in heterogeneous scenarios, especially in scenarios 3 and 4, where the MSEs for nugget subgroups exceed 0.4. This is already more than four times that of the other two methods. The bias of BHM is also large in heterogeneous scenarios, and the absolute values of the bias for some subgroups are higher than 0.2 (Fig. 4), while the IBIS is much more robust. Notably, although BHM borrows information across all subgroups, the 95% CIs for its estimates are generally wider in heterogeneous scenarios than that of IBIS. This is because the variance across subgroups ${\overset{\sim }{\sigma}}^2$ estimated by BHM is large due to heterogeneous subgroups, resulting in limited information borrowing. In contrast, the subgroup division in IBIS makes the similarity within each subset higher and thus leads to more information borrowing.

In summary, from the three metrics (MSE, bias and 95% CI width), the estimation performance of the IBIS is superior and robust under the pre-specified scenarios. The proposed method is especially suitable for scenarios where subgroups should be divided into two subsets.

### Operating characteristics of subgroup identification

The simulation results of the operating characteristics are presented in Table 3. In the global alternative scenario (scenario 2), BHM can identify all subgroups correctly with a 100% probability, indicating a strong borrowing strength. The Freq method achieves nearly 90% conjunctive power (hereinafter referred to as power), which reflects the advantage of partial combination. The power produced by IBIS is slightly lower than that of the Freq method but is much higher than that of the independent analysis.

**Table 3:** Operating characteristics of four methods under eight scenarios

	Simulation 1		Simulation 2	
Method	FWER	Conjunctive Power	FWER	Conjunctive Power
	*Scenario* 1		*Scenario* 1	
IBIS	0.0971	/	0.0490	/
Independent	0.1000		0.0496	
BHM	0.0991		0.0500	
Freq	0.0985		0.0499	
	*Scenario* 2		*Scenario* 2	
IBIS	/	0.8602	/	0.8092
Independent		0.6089		0.4664
BHM		1.0000		1.0000
Freq		0.8961		0.8787
	*Scenario* 3		*Scenario* 3	
IBIS	0.0455	0.8984	0.0265	0.8193
Independent	0.0916	0.6447	0.0449	0.4910
BHM	0.3093	0.6027	0.1760	0.4587
Freq	0.2149	0.8609	0.1717	0.7582
	*Scenario* 4		*Scenario* 4	
IBIS	0.0578	0.9541	0.0399	0.9341
Independent	0.0079	0.3748	0.0045	0.2251
BHM	0.7718	0.9980	0.6805	0.9964
Freq	0.1072	0.8101	0.0866	0.7900
	*Scenario* 5		*Scenario* 5	
IBIS	0.1586	0.8774	0.1106	0.8393
Independent	0.0693	0.6200	0.0343	0.4743
BHM	0.6445	0.9768	0.4459	0.9407
Freq	0.1772	0.8480	0.1647	0.8375
	*Scenario* 6		*Scenario* 6	
IBIS	0.1178	0.8092	0.0838	0.7779
Independent	0.0349	0.3766	0.0175	0.2201
BHM	0.7028	0.9891	0.5440	0.9686
Freq	0.1153	0.7656	0.0973	0.7508
	*Scenario* 7		*Scenario* 7	
IBIS	0.1877	0.8108	0.1508	0.7742
Independent	0.0525	0.2507	0.0248	0.1185
BHM	0.6700	0.9688	0.4945	0.9184
Freq	0.1860	0.7138	0.1712	0.7012
	*Scenario* 8		*Scenario* 8	
IBIS	0.0843	0.8617	0.0386	0.7637
Independent	0.0079	0.4113	0.0045	0.2171
BHM	0.4294	0.9982	0.3169	0.9937
Freq	0.3961	0.9030	0.3030	0.8597

Simulations 1 & 2 correspond to different degrees of control over FWER in the global null scenario (scenario 1)

For global null scenario (scenario 1), calculating the conjunctive power makes no sense. Similarly, for global alternative scenario (scenario 2), calculating the FWER makes no sense

When the subgroups are heterogeneous (scenarios 3–8), the IBIS outperforms the other three methods, evidenced by a better balance between FWER and power. Compared with independent analysis, the power of the IBIS is much higher. When the investigational therapy is effective for several subgroups (scenarios 4, 6, 7, and 8), it is difficult for independent analysis to identify all alternative subgroups correctly at the same time. The FWER of IBIS is slightly higher than that of independent analysis in most scenarios, except in scenario 3, where information borrowing across low-efficacy subgroups reduces the type I error rates. However, such type I error inflation is relatively limited, for example, approximately 10–20% in scenarios 5–7. At this time, the number of null subgroups is small, and the borrowing strength between subgroups is not as strong as that in scenarios 1–4. Therefore, there are some misjudgments for null subgroups. There is a consensus that controlling type I error rates is critical in clinical trials. But it should also be recognized that if the investigational drug is effective for a large portion of the population (e.g., scenario 5), it may be inappropriate to borrow no information at all. In scenarios 5–7, although FWER inflates, the proposed IBIS design misjudge only one or two null subgroups in most cases (see section A of the Additional file 1 for the the detailed simulated probabilities of misjudging null and alternative subgroups in each scenario). Taking scenario 5 in simulation 1 as an example, there are a total of 8 subgroups with low efficacy to treatment, and the family-wise type I error rate is 15.86%, higher than 10%. But most of the error cases are misjudging one (9.52%) or two subgroups (4.54%). In these cases, the investigational drug combination are effective in 2/3 of the identified subgroups and are ineffective in 1/3 of the identified subgroups. Therefore, given that some marketed drugs are not effective for all patients, this kind of type I error inflation is acceptable to some extent. We believe that strictly controlling the type I error of one or two subgroups at the expense of power gains of most subgroups is not desirable for subgroup identification in an exploratory trial.

The power produced by BHM is high in most scenarios, except in scenario 3, where there is only one good nugget subgroup. However, BHM also tends to overestimate the efficacy of investigational therapy for low-efficacy subgroups, resulting in unacceptable FWERs.

The operating characteristics of the Freq method are relatively robust compared with independent analysis and BHM, but are inferior to the proposed IBIS in general. This is due to its strategy of partial combination. For example, if Freq mistakenly classifies an alternative subgroup into a low-efficacy subset, it is likely to accept the null hypothesis in subsequent inferences and conclude that the investigational therapy is ineffective for all subgroups in the low-efficacy subset. In contrast, the IBIS method, although also making subgroup divisions, only borrows information within each subset, and the inferences are still based on each subgroup. Therefore, the probabilities of making such incorrect decisions are smaller in most scenarios.

Additional sensitivity analyses evaluating the prior settings for the shrinkage parameters are performed and the results are shown in section B of the Additional file 1. The results show that the used inverse-gamma priors are robust in our simulation study. But it should be noted that there may be no optimal choice for the prior distributions. The priors we used here may be not applicable in other studies. The appropriate prior distributions for a particular trial must be determined by the cooperation of clinicians and statisticians at the time of trial design. See section B of the Additional file 1 for more details and remarks.

### An example of how to obtain design parameters for a multi-stage adaptive enrichment design

**Fig. 6:**
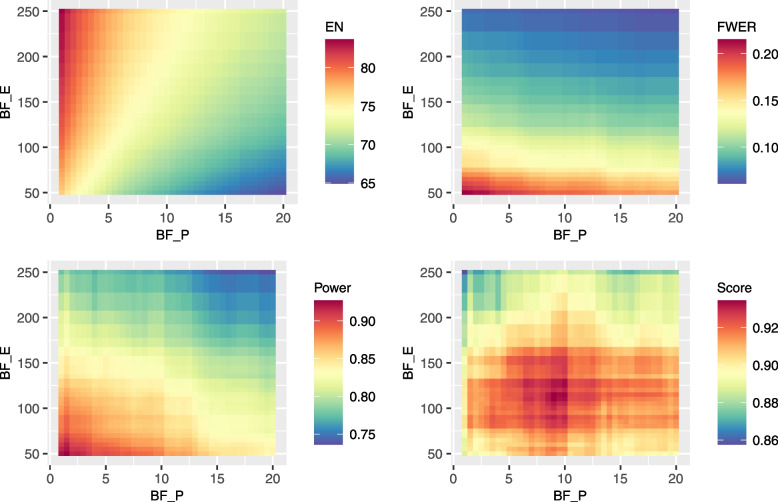
Operating characteristics of the two-stage adaptive enrichment design with varying decision thresholds under scenario 8. EN, expected sample size; FWER, family-wise type I error rate; Power, conjunctive power; Score, values of decision score

Regarding the adaptive enrichment design, we evaluate the performance of IBIS under different values of *BF*_*E*_ and *BF*_*P*_ for scenario 8 and draw corresponding heatmaps for FWER, conjunctive power, expected sample size and decision score (Fig. 6). The corresponding heatmaps for other scenarios are shown in section C of the Additional file 1. With the help of such heatmaps, decision-makers can more intuitively observe the changes of the above metrics along with the decision thresholds and thus make trade-offs between these metrics.

It should be noted that the restriction ${BF}_{E\left(k,j\right)}^{(1)}={BF}_{E\left(k,j\right)}^{(2)}={BF}_E$ and ${BF}_{P\left(k,j\right)}^{(1)}={BF}_P$ is set mainly because we want to first have a general overview of the operating characteristics with different combinations of decision thresholds. After determining the range of decision thresholds that meet the requirements (in the example above, when *BF*_*P*_ is from 5.0 to 12.5 and *BF*_*E*_ is from 75 to 150, the decision score is relatively high), we can further adjust such thresholds according to the anticipated efficacy and prevalence of each subgroup.

In addition to FWER, conjunctive power and expected sample size, some other metrics can also be considered, such as the disjunctive power, which is defined as follows:

$$Disjunctive\ Power=\Pr \left( Reject\ at\ least\ one\ {H}_{0\left(k,j\right)}|{H}_{1\left(k,j\right)}\ is\ true\right)$$

This metric makes sense, especially when the sample size is limited. On the other hand, the operating characteristics of the design in other scenarios should be also taken into account (see section C of the Additional file 1). In conclusion, the composition of the decision score function and the weights of different metrics need to be discussed sufficiently by researchers, sponsors and biostatisticians. We should set up reasonable scenarios based on existing medical research data case-by-case and take into account the requirements of various stakeholders when calibrating parameters of multi-stage adaptive enrichment designs.

## Discussion

Compared with monotherapy, targeted combination therapy leads to an increasing number of subgroups and complicated biomarker-based efficacy profiles. Therefore, we propose a statistical tool called IBIS in this article and incorporate it into the adaptive enrichment design framework. The IBIS contains three main elements: subgroup division, efficacy evaluation and subgroup identification. We first enumerate all possible subgroup divisions based on biomarker levels. Then, Jensen–Shannon divergence is used to distinguish high-efficacy and low-efficacy subgroups, and BHM is employed to borrow information within these two subsets for efficacy evaluation. Regarding subgroup identification, a hypothesis testing framework based on Bayes factors, which also plays a key role in go/no-go decisions and enriching specific subgroups in subsequent stage of trial, is constructed. Simulation studies show that, compared with some traditional methods, our proposed IBIS has superior and robust operating characteristics, and has the potential to be a useful tool for subgroup identification and population enrichment in clinical trials of targeted combination therapy.

As the combination of two targeted agents is a promising therapeutic approach at present, we take trial designs that consider two ordinal biomarkers as examples in this paper. The proposed design can be naturally extended for subgroup identification where multiple biomarkers are incorporated. We can still enumerate all possible subgroup divisions, find the optimal division, and then use the BHM to borrow information within each subset. In terms of continuous biomarkers, if we cannot pre-define subgroups and the study objective is to find arbitrary biomarker cutoffs, the proposed design cannot work. If the subgroups can be pre-defined, e.g., be divided by quartiles, then the proposed design can still be applied.

Another feasible monitoring method for interim analysis is to calculate a predictive probability that *BF*_(*k*, *j*)_ exceed a success threshold at the end of the trial. Each time an interim analysis is performed, this predictive probability is compared with pre-specified cutoffs to decide the target population in the subsequent stage. Some researches, e.g., Lee and Liu (2008) [30], found that comparable operating characteristics can be obtained by taking both the predictive probability and the posterior probability approaches. This conclusion should also be generalized to our proposed design as the predictive probability approach does not include more patients’ data compared with the posterior probability approach. One advantage of the predictive probability approach is that it can give the probability of finally obtaining a positive result at interim analysis. However, it also requires more computational expense when predicting future data.

Some points need to be paid attention to when applying IBIS. For example, when the number of subgroups is small, it may be inappropriate to directly use the inverse gamma distribution with small shape and scale parameters as a prior for the variances across subgroups [31]. Some weakly informative priors can be applied instead. The prevalence of each subgroup also influences trial design and conduction. Enrollment of patients may be difficult when the prevalence of certain subgroups is low, and we cannot accumulate enough evidence to demonstrate how well the investigational therapy works on those subgroups. On the other hand, the treatment parameters of subgroups with smaller sample sizes tend to have stronger shrinkage in BHM [32], potentially causing misleading interpretations of trial results. When faced with such a problem, our suggestion is to first consider whether the anticipated efficacy for the rare subgroups is close to that of the adjacent subgroups based on clinical experience. If they are close enough, pooled analysis is suggested; if it is uncertain whether they are close, an independent exploratory analysis for the rare subgroups is recommended rather than a BHM-based analysis.

Regarding the choice between single-arm trial and RCT, we must admit that RCT is a better choice when cost, ethics and some other aspects allow, due to its advantages of eliminating selection bias and minimizing confoundings. However, RCT also has the limitations of high cost, long period and difficult implementation, which may not be appropriate if such a subgroup identification or enrichment trial is positioned as an exploratory trial. One strategy that deserves further exploration is the seamless transition design from open-label single-arm to randomized double-arm clinical trials.

There are also some other areas where this research can be extended. For example, the sample size re-estimation based on the Bayes factors can be considered in the interim analysis. Historical information on monotherapy can be incorporated to determine the priors in Bayes factors. There is also a class of adaptive enrichment designs that take into account situations where subgroups are not pre-defined [33–42]. Therefore, in addition to multi-level biomarkers, we can further consider how to determine the cutoffs of continuous biomarkers, or include other important covariates in the model, such as age, Eastern Cooperative Oncology Group Performance Status (ECOG PS), key laboratory testing results, etc., which is more in line with the concept of precision medicine.

## Conclusions

IBIS has superior and robust operating characteristics in terms of subgroup identification and population enrichment. It has the potential to be a useful tool for biomarker-based subgroup identification in clinical trials of targeted antitumor combination therapy.

## Supplementary Information

**Additional file 1.** Supplemental simulation results.

## Data Availability

The R code for simulation during the current study are available in the github repository, https://github.com/cccc633/IBIS.

## Abbreviations

BHM: Bayesian hierarchical model
ADC: antibody-drug conjugate
RCT: randomized controlled trial
FWER: family-wise error rate
MSE: mean squared error
ECOG PS: Eastern Cooperative Oncology Group Performance Status
