# Supplementary material for: IBIS: identify biomarker-based subgroups with a Bayesian enrichment design for targeted combination therapy
Source: BMC Med Res Methodol. 2023 Mar 20;23:66. doi: 10.1186/s12874-023-01877-w (PMC10026491; doi:10.1186/s12874-023-01877-w)
Supplement: Supplementary file 1 — Additional file 1. Supplemental simulation results. [file 12874_2023_1877_MOESM1_ESM.pdf]

Additional file 1

Supplemental simulation results

A. Supplemental simulation results for the subgroup identification

This part displays supplemental operating characteristics of four methods under eight scenarios for subgroup identification. We give the simulated probabilities of misjudging null and alternative subgroups in detail. The family-wise type I error rate and conjunctive power are referred to as FWER and Power, respectively. Simulations 1 and 2 correspond to different degrees of control over FWER in scenario 1.

Table A1 The percentages of misjudging null subgroups as effective in scenario 1.

Simulation 1													
Method	FWER	The number of null subgroups that are misjudged as effective											
		1	2	3	4	5	6	7	8	9	10	11	12
IBIS	9.71	4.73	1.85	1.21	.76	.14	.47	.08	.17	.13	.00	.02	.15
Independent	10.00	.84	1.53	1.68	1.48	.00	1.87	.01	.92	.87	.01	.00	.79
BHM	9.91	.31	.57	.63	.63	.12	1.19	.18	.93	1.15	.62	.49	3.09
Freq	9.85	1.79	1.22	1.50	1.12	.78	.92	.54	.73	.48	.24	.15	.38
Simulation 2													
Method	FWER	The number of null subgroups that are misjudged as effective											
		1	2	3	4	5	6	7	8	9	10	11	12
IBIS	4.90	2.86	.84	.50	.33	.00	.15	.03	.04	.07	.00	.00	.08
Independent	4.96	.47	.73	.95	.73	.00	.73	.01	.46	.43	.00	.00	.45
BHM	5.00	.11	.29	.33	.39	.08	.68	.13	.63	.63	.20	.13	1.40
Freq	4.99	1.12	.61	.80	.53	.34	.45	.26	.33	.20	.11	.07	.17

Table A2 The percentages of misjudging alternative subgroups as ineffective in scenario 2.

Simulation 1													
Method	Power	The number of alternative subgroups that are misjudged as ineffective											
		1	2	3	4	5	6	7	8	9	10	11	12
IBIS	86.02	11.65	1.94	.37	.02	.00	.00	.00	.00	.00	.00	.00	.00
Independent	60.89	14.10	11.63	6.98	3.81	1.33	.86	.21	.13	.04	.02	.00	.00
BHM	100.00	.00	.00	.00	.00	.00	.00	.00	.00	.00	.00	.00	.00
Freq	89.61	8.29	1.92	.18	.00	.00	.00	.00	.00	.00	.00	.00	.00

Table A2 (continued)

Simulation 2

Method	Power	The number of alternative subgroups that are misjudged as ineffective											
		1	2	3	4	5	6	7	8	9	10	11	12
IBIS	80.92	14.28	3.89	.77	.14	.00	.00	.00	.00	.00	.00	.00	.00
Independent	46.64	11.78	12.44	11.82	8.00	3.37	3.47	1.05	.75	.32	.22	.07	.07
BHM	100.00	.00	.00	.00	.00	.00	.00	.00	.00	.00	.00	.00	.00
Freq	87.87	8.50	3.20	.42	.01	.00	.00	.00	.00	.00	.00	.00	.00

Table A3 The percentages of misjudging null (alternative) subgroups as effective (ineffective) in scenario 3.

Simulation 1												
Method	FWER	The number of null subgroups that are misjudged as effective										
		1	2	3	4	5	6	7	8	9	10	11
IBIS	4.55	1.83	1.18	.59	.14	.45	.08	.11	.08	.01	.01	.07
Independent	9.16	1.53	1.68	1.48	.00	1.87	.01	.92	.87	.01	.00	.79
BHM	30.93	2.28	2.81	2.49	.53	4.33	.87	3.59	3.70	1.41	1.23	7.69
Freq	21.49	9.79	4.40	2.63	.91	1.26	.62	.50	.44	.23	.16	.55
Method	Power	The number of alternative subgroups that are misjudged as ineffective										
		1										
IBIS	89.84	10.16										
Independent	64.47	35.53										
BHM	60.27	39.73										
Freq	86.09	13.91										
Simulation 2												
Method	FWER	The number of null subgroups that are misjudged as effective										
		1	2	3	4	5	6	7	8	9	10	11
IBIS	2.65	1.38	.73	.27	.01	.17	.03	.03	.01	.00	.00	.02
Independent	4.49	.73	.95	.73	.00	.73	.01	.46	.43	.00	.00	.45
BHM	17.60	1.52	1.88	1.78	.26	2.97	.38	1.87	2.13	.65	.40	3.76
Freq	17.17	8.18	3.45	2.03	.76	.93	.51	.43	.32	.16	.12	.28
Method	Power	The number of alternative subgroups that are misjudged as ineffective										
		1										
IBIS	81.93	18.07										
Independent	49.10	50.90										
BHM	45.87	54.13										
Freq	75.82	24.18										

Table A4 The percentages of misjudging null (alternative) subgroups as effective (ineffective) in scenario 4.

Simulation 1												
Method	FWER	The number of null subgroups that are misjudged as effective										
		1										
IBIS	5.78	5.78										
Independent	.79	.79										
BHM	77.18	77.18										
Freq	10.72	10.72										
Method	Power	The number of alternative subgroups that are misjudged as ineffective										
		1	2	3	4	5	6	7	8	9	10	11
IBIS	95.41	3.32	1.11	.11	.04	.01	.00	.00	.00	.00	.00	.00
Independent	37.48	28.72	18.23	9.47	3.10	2.07	.57	.27	.05	.04	.00	.00
BHM	99.80	.19	.01	.00	.00	.00	.00	.00	.00	.00	.00	.00
Freq	81.01	14.26	3.97	.74	.02	.00	.00	.00	.00	.00	.00	.00
Simulation 2												
Method	FWER	The number of null subgroups that are misjudged as effective										
		1										
IBIS	3.99	3.99										
Independent	.45	.45										
BHM	68.05	68.05										
Freq	8.66	8.66										
Method	Power	The number of alternative subgroups that are misjudged as ineffective										
		1	2	3	4	5	6	7	8	9	10	11
IBIS	93.41	4.59	1.55	.29	.13	.03	.00	.00	.00	.00	.00	.00
Independent	22.51	22.81	22.30	14.87	5.95	6.71	2.06	1.57	.68	.30	.13	.11
BHM	99.64	.35	.01	.00	.00	.00	.00	.00	.00	.00	.00	.00
Freq	79.00	14.85	4.84	1.22	.08	.01	.00	.00	.00	.00	.00	.00

Table A5 The percentages of misjudging null (alternative) subgroups as effective (ineffective) in scenario 5.

Simulation 1									
Method	FWER	The number of null subgroups that are misjudged as effective							
		1	2	3	4	5	6	7	8
IBIS	15.86	9.52	4.54	.66	.53	.28	.05	.04	.24
Independent	6.93	1.68	2.64	.02	.92	.87	.01	.00	.79
BHM	64.45	7.12	12.73	2.81	7.03	8.98	3.78	2.91	19.09
Freq	17.72	9.90	3.68	.71	.38	.19	.05	.01	2.80

Method	Power	The number of alternative subgroups that are misjudged as ineffective			
		1	2	3	4
IBIS	87.74	6.22	2.53	3.46	.05
Independent	62.00	14.41	17.72	3.70	2.17
BHM	97.68	1.82	.35	.06	.09
Freq	84.80	8.60	5.36	1.22	.02

Simulation 2									
Method	FWER	The number of null subgroups that are misjudged as effective							
		1	2	3	4	5	6	7	8
IBIS	11.06	7.36	2.91	.32	.33	.09	.02	.00	.03
Independent	3.43	.95	1.13	.01	.46	.43	.00	.00	.45
BHM	44.59	6.38	11.10	1.72	5.48	6.27	1.73	1.50	10.41
Freq	16.47	9.95	3.69	.71	.38	.19	.05	.01	1.49

Method	Power	The number of alternative subgroups that are misjudged as ineffective			
		1	2	3	4
IBIS	83.93	7.43	3.42	5.02	.20
Independent	47.43	11.57	25.97	7.26	7.77
BHM	94.07	4.29	1.16	.17	.31
Freq	83.75	8.84	5.81	1.58	.02

Table A6 The percentages of misjudging null (alternative) subgroups as effective (ineffective) in scenario 6.

Simulation 1					
Method	FWER	The number of null subgroups that are misjudged as effective			
		1	2	3	4
IBIS	11.78	5.55	4.13	1.16	.94
Independent	3.49	.90	1.80	.00	.79
BHM	70.28	6.96	20.15	6.75	36.42
Freq	11.53	4.60	1.80	.15	4.98

Method	Power	The number of alternative subgroups that are misjudged as ineffective							
		1	2	3	4	5	6	7	8
IBIS	80.92	13.57	4.60	.70	.20	.01	.00	.00	.00
Independent	37.66	23.08	23.97	8.77	4.38	1.45	.55	.11	.03
BHM	98.91	1.04	.05	.00	.00	.00	.00	.00	.00
Freq	76.56	15.14	7.00	1.03	.24	.03	.00	.00	.00

Simulation 2					
Method	FWER	The number of null subgroups that are misjudged as effective			
		1	2	3	4
IBIS	8.38	4.68	2.93	.37	.40
Independent	1.75	.41	.89	.00	.45
BHM	54.40	6.92	17.90	4.48	25.10
Freq	9.73	4.62	1.80	.15	3.16

Method	Power	The number of alternative subgroups that are misjudged as ineffective							
		1	2	3	4	5	6	7	8
IBIS	77.79	15.09	5.58	1.05	.40	.05	.00	.04	.00
Independent	22.01	17.19	28.62	13.40	10.70	4.73	2.14	.60	.61
BHM	96.86	2.90	.23	.01	.00	.00	.00	.00	.00
Freq	75.08	15.47	7.73	1.29	.35	.06	.01	.01	.00

Table A7 The percentages of misjudging null (alternative) subgroups as effective (ineffective) in scenario 7.

Simulation 1							
Method	FWER	The number of null subgroups that are misjudged as effective					
		1	2	3	4	5	6
IBIS	18.77	15.74	1.25	1.08	.19	.17	.34
Independent	5.25	2.66	.00	1.79	.01	.00	.79
BHM	67.00	14.19	3.11	15.37	4.66	4.32	25.35
Freq	18.60	12.75	1.39	.57	.07	.03	3.79
Method	Power	The number of alternative subgroups that are misjudged as ineffective					
		1	2	3	4	5	6
IBIS	81.08	16.13	2.12	.48	.06	.13	.00
Independent	25.07	42.17	19.45	9.61	2.84	.56	.30
BHM	96.88	3.03	.08	.01	.00	.00	.00
Freq	71.38	23.38	3.45	1.53	.17	.09	.00
Simulation 2							
Method	FWER	The number of null subgroups that are misjudged as effective					
		1	2	3	4	5	6
IBIS	15.08	13.35	.76	.76	.08	.04	.09
Independent	2.48	1.14	.00	.89	.00	.00	.45
BHM	49.45	13.48	2.28	13.06	2.75	2.32	15.56
Freq	17.12	12.77	1.39	.57	.07	.03	2.29
Method	Power	The number of alternative subgroups that are misjudged as ineffective					
		1	2	3	4	5	6
IBIS	77.42	18.60	2.74	.75	.17	.31	.01
Independent	11.85	34.17	25.06	17.54	7.35	1.92	2.11
BHM	91.84	7.67	.44	.05	.00	.00	.00
Freq	70.12	23.95	3.69	1.80	.30	.14	.00

Table A8 The percentages of misjudging null (alternative) subgroups as effective (ineffective) in scenario 8.

Simulation 1							
Method	FWER	The number of null subgroups that are misjudged as effective					
		1					
IBIS	8.43	8.43					
Independent	.79	.79					
BHM	42.94	42.94					
Freq	39.61	39.61					
Method	Power	The number of alternative subgroups that are misjudged as ineffective					
		1	2	3	4	5	6
IBIS	86.17	12.01	1.64	.17	.01	.00	.00
Independent	41.13	38.47	14.63	4.62	1.03	.12	.00
BHM	99.82	.18	.00	.00	.00	.00	.00
Freq	90.30	8.76	.84	.10	.00	.00	.00
Simulation 2							
Method	FWER	The number of null subgroups that are misjudged as effective					
		1					
IBIS	3.86	3.86					
Independent	.45	.45					
BHM	31.69	31.69					
Freq	30.30	30.30					
Method	Power	The number of alternative subgroups that are misjudged as ineffective					
		1	2	3	4	5	6
IBIS	76.37	19.01	3.80	.70	.11	.01	.00
Independent	21.71	36.50	24.58	11.61	4.53	.92	.15
BHM	99.37	.62	.01	.00	.00	.00	.00
Freq	85.97	12.33	1.49	.21	.00	.00	.00

B. Sensitivity analysis of prior settings for the shrinkage parameters

This document displays the simulation results of sensitivity analysis. We evaluate the performance of IBIS for subgroup identification when setting various prior distributions for the shrinkage parameters σ_g^2 and σ_{Cg}^2. In the main document, their priors are both set as an inverse-gamma distribution $IG(0.001,0.001)$. Here, we further consider the following settings:

(1) A uniform prior distribution on a very wide interval $[0,1000]$, which is a typical noninformative prior.

(2) Half-normal distributions are also used as the priors of shrinkage parameters in some papers, e.g., Neuenschwander et al. (2010) and Schmidli et al. (2014). We first consider a half-normal distribution $HN(0,1000)$ with mean 0 and variance 1000, which is nearly noninformative. As the intersubgroup variances for all 12 simulation scenarios are no more than 0.28, we further set $HN(0,0.25)$ and $HN(0,0.0625)$ as the prior distributions.

(3) An inverse-gamma prior $IG(0.0005,0.000005)$ proposed in a previous research by Berry et al. (2013).

(4) Another inverse-gamma distribution with small shape parameter and scale parameter $IG(0.01,0.01)$, which has been used in Jin and Yin (2021).

The simulation results are shown in Figure A2-1 and A2-2. It can be seen that, when setting $Unif(0,1000)$ or $HN(0,1000)$ as the prior distributions for the shrinkage parameters, the design tends to borrow less information across subgroups and thus results in lower family-wise type I error rate (FWER) and lower conjunctive power (hereinafter referred to as power). However, as the intersubgroup variances for all 12 simulation scenarios are no more than 0.28, these two priors may seem too conservative. When the half-normal prior distributions become more concentrated at 0, i.e., the $HN(0,0.25)$ and $HN(0,0.0625)$, the design would produce higher FWER and power.

In terms of the inverse-gamma priors with small shape parameter and scale parameter, the amount of borrowing would be strong and thus the power can be improved. However, they also result in relatively higher FWER, especially when we

set extreme small shape parameter and scale parameter for the inverse-gamma distribution, i.e., $IG(0.0005,0.000005)$. The priors $IG(0.001,0.001)$ and $IG(0.01,0.01)$ are more robust in this situation.

Generally speaking, in our simulation study, it is not recommended to use $Unif(0,1000)$, $HN(0,1000)$ or $IG(0.0005,0.000005)$ as the priors for σ_g^2 and σ_{Cg}^2. The first two borrow little information and the improvements on power may be limited in some scenarios, while the last one may induce very high FWER in some scenarios. For priors $IG(0.001,0.001)$, $IG(0.01,0.01)$, $HN(0,0.25)$ and $HN(0,0.0625)$, the performances of the proposed design are similar. In order to more distinctly show the influence of different priors on the strength of borrowing, we also evaluate the estimation performance. The performance when using $HN(0,1000)$ and $HN(0,0.25)$ are similar to that of $Unif(0,1000)$ and $HN(0,0.0625)$, respectively. To make it easier for readers to identify the elements in the figures, these two cases are omitted. The results (Figure A2-3 to A2-5) are indeed consistent with what we have stated before. The uniform prior $Unif(0,1000)$ corresponds higher mean squared errors (MSE) and wider widths of the 95% incredible interval (95% CI), indicating less information borrowing. The inverse-gamma prior $IG(0.0005,0.000005)$ is in the opposite way, indicating strong borrowing. As for both the MSE and bias (Figure A2-3 and A2-4), the priors $IG(0.001,0.001)$, $IG(0.01,0.01)$ and $HN(0,0.0625)$ are more robust than $Unif(0,1000)$ and $IG(0.0005,0.000005)$.

It should be noted that there may be no optimal choice for the prior distributions. Although some priors demonstrated above can perform well in our simulations, they may be not applicable in other studies. The appropriate prior distributions for a particular trial must be determined by the cooperation of clinicians and statisticians at the time of trial design.

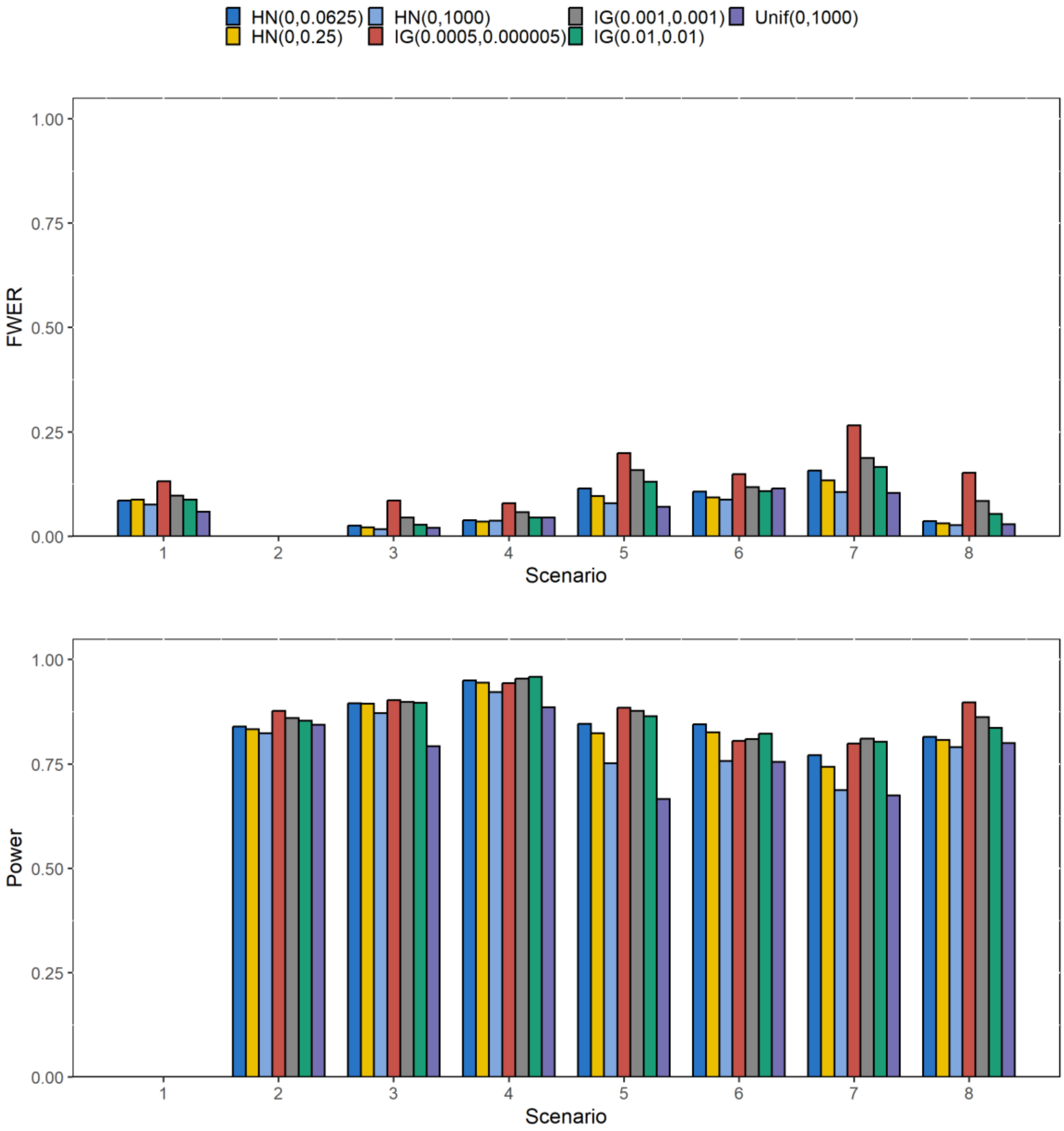

Fig. B1 Operating characteristics of the IBIS design for subgroup identification when setting different priors for the shrinkage parameters. The threshold are the same as that used in Simulation 1 of the main document. FWER denotes the family-wise type I error rate and Power denotes the conjunctive power. The label 'IG(α,β)' denotes an inverse-gamma distribution with a shape parameter α and a scale parameter β; The label 'HN(μ,σ^2)' denotes a half-normal distribution with mean μ and variance σ^2; The label Unif(α,β) denotes a uniform distribution on the interval [α,β].

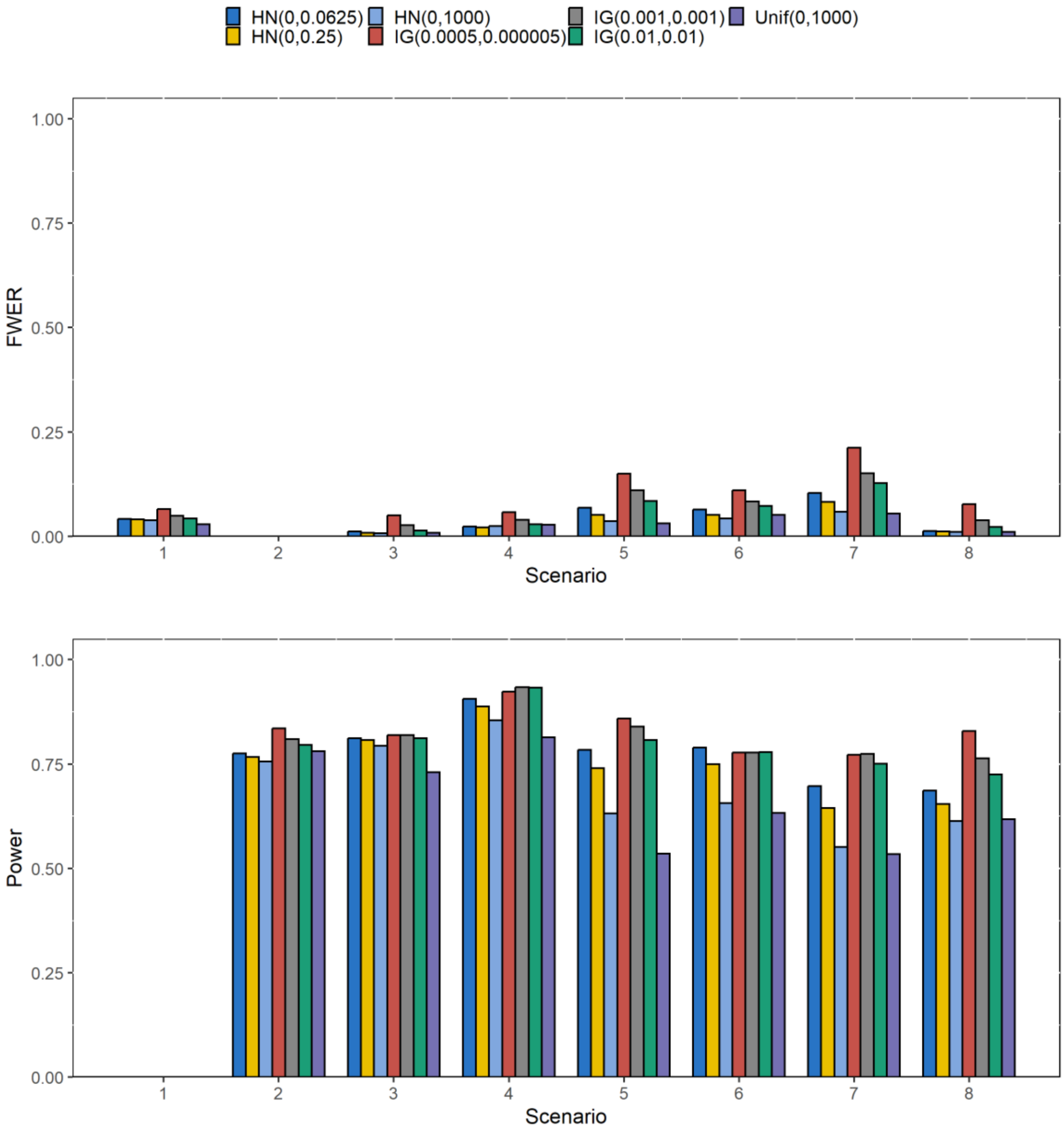

Fig. B2 Operating characteristics of the IBIS design for subgroup identification when setting different priors for the shrinkage parameters. The threshold are the same as that used in Simulation 2 of the main document. FWER denotes the family-wise type I error rate and Power denotes the conjunctive power. The label 'IG(α,β)' denotes an inverse-gamma distribution with a shape parameter α and a scale parameter β; The label 'HN(μ,σ^2)' denotes a half-normal distribution with mean μ and variance σ^2; The label Unif(α,β) denotes a uniform distribution on the interval [α,β].

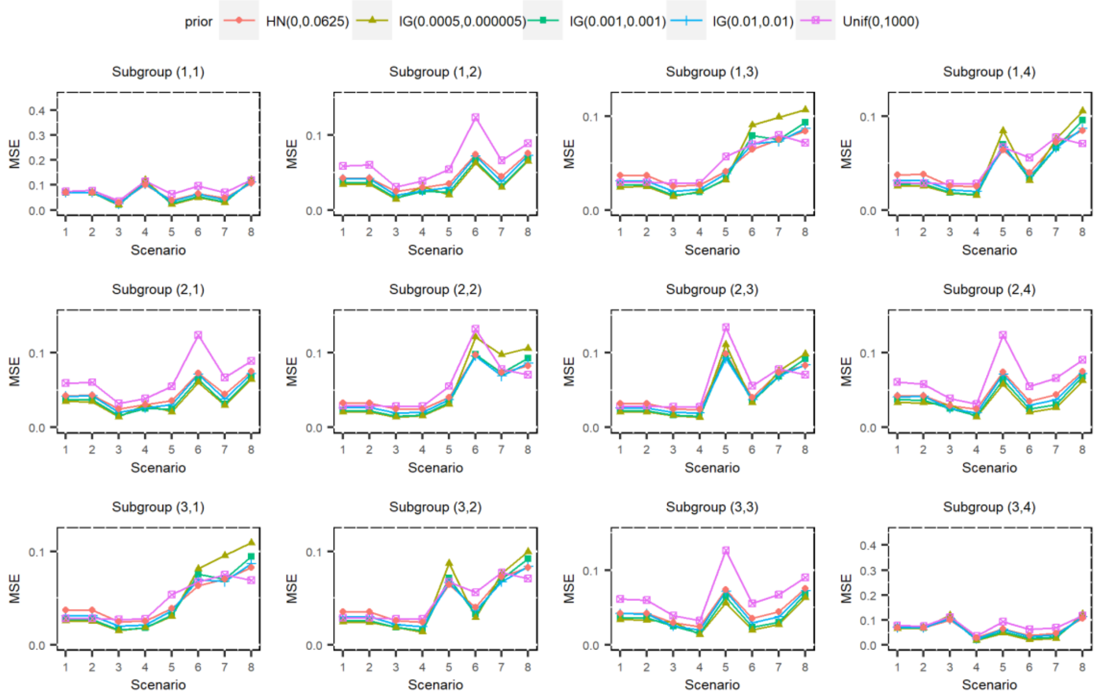

Fig. B3 Simulated mean squared error (MSE) of treatment effect estimates for 12 subgroups under eight scenarios using 'IBIS' when setting different priors for the shrinkage parameters. The label 'IG(α,β)' denotes an inverse-gamma distribution with a shape parameter α and a scale parameter β; The label 'HN(μ,σ^2)' denotes a half-normal distribution with mean μ and variance σ^2; The label Unif(α,β) denotes a uniform distribution on the interval [α,β].

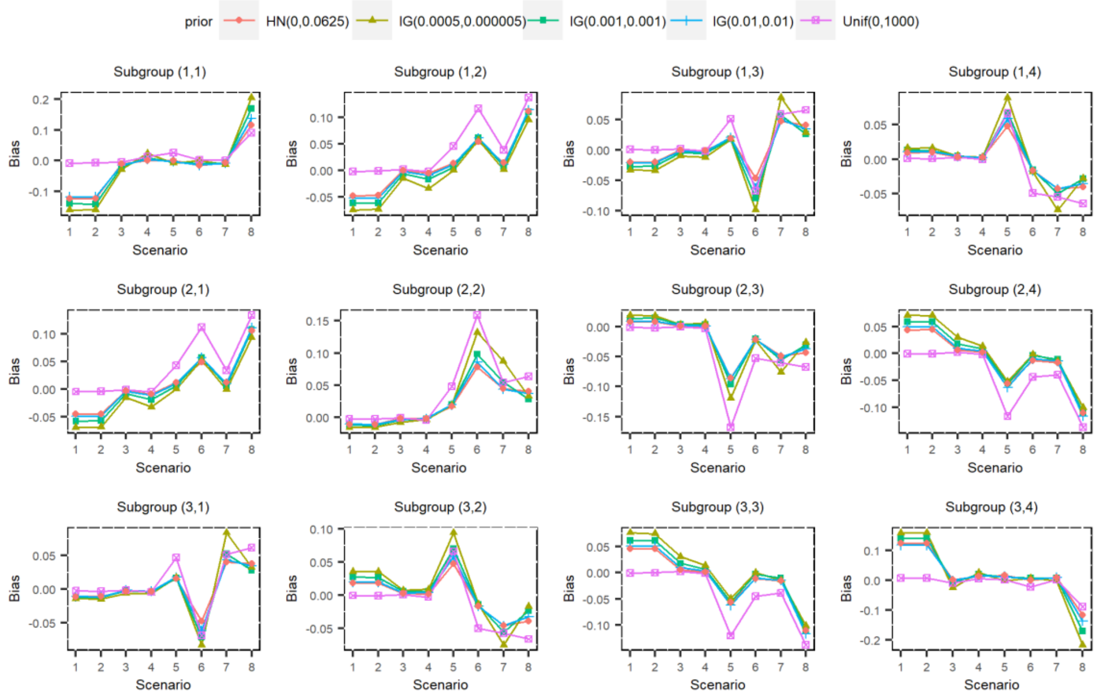

Fig. B4 Simulated bias of treatment effect estimates for 12 subgroups under eight scenarios using 'IBIS' when setting different priors for the shrinkage parameters. The label 'IG(α,β)' denotes an inverse-gamma distribution with a shape parameter α and a scale parameter β; The label 'HN(μ,σ^2)' denotes a half-normal distribution with mean μ and variance σ^2; The label Unif(α,β) denotes a uniform distribution on the interval [α,β].

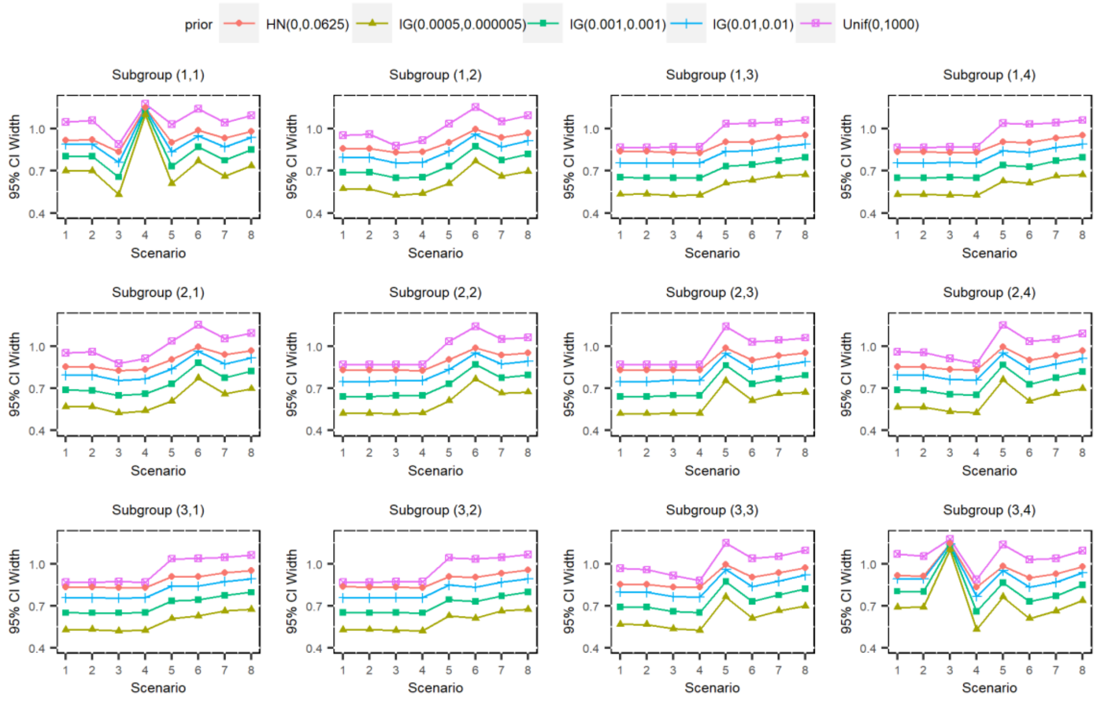

Fig. B5 Simulated average width of 95% credible interval of treatment effect estimates for 12 subgroups under eight scenarios using 'IBIS' when setting different priors for the shrinkage parameters. The label 'IG(α,β)' denotes an inverse-gamma distribution with a shape parameter α and a scale parameter β; The label 'HN(μ,σ^2)' denotes a half-normal distribution with mean μ and variance σ^2; The label Unif(α,β) denotes a uniform distribution on the interval [α,β].

References

1. Berry SM, Broglio KR, Groshen S, Berry DA. Bayesian hierarchical modeling of patient subpopulations: efficient designs of phase II oncology clinical trials. Clin Trials. 2013;10(5): 720-734.
2. Jin H, Yin G. Unit information prior for adaptive information borrowing from multiple historical datasets. Stat Med. 2021;40(25):5657-5672.
3. Neuenschwander B, Capkun-Niggli G, Branson M, Spiegelhalter DJ. Summarizing historical information on controls in clinical trials. Clin Trials. 2010;7(1):5-18.
4. Schmidli H, Gsteiger S, Roychoudhury S, O'Hagan A, Spiegelhalter D, Neuenschwander B. Robust meta-analytic-predictive priors in clinical trials with historical control information. Biometrics. 2014;70(4):1023-1032.

C. Supplemental simulation results for the two-stage enrichment design

This document displays supplemental operating characteristics of the two-stage adaptive enrichment design with varying decision thresholds. Similar to Figure 6 in the main document, we draw heatmaps for FWER, conjunctive power, expected sample size and decision score in scenarios 1-7. For global null scenario (scenario 1), calculating the conjunctive power makes no sense. Therefore, calculating the decision score also becomes unnecessary. What we primarily concerned with is the FWER and expected sample size. Similarly, for global alternative scenario (scenario 2), calculating the FWER makes no sense. So there are only two subfigures for scenario 1 and scenario 2.

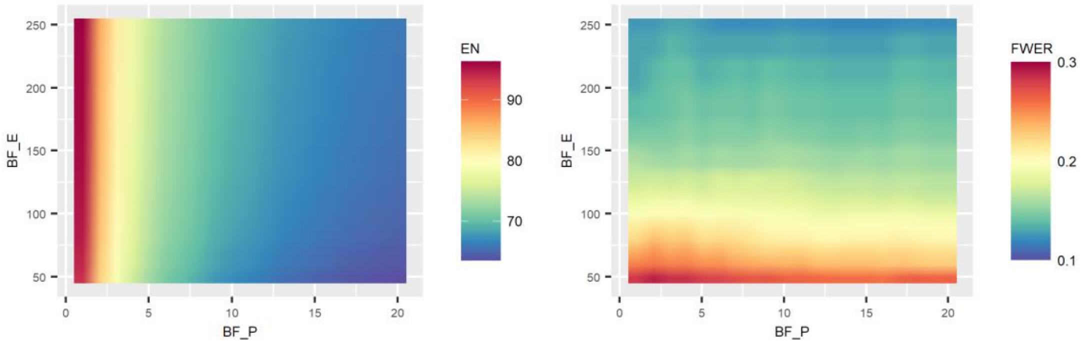

Fig. C1 Operating characteristics of the two-stage adaptive enrichment design with varying decision thresholds under scenario 1.

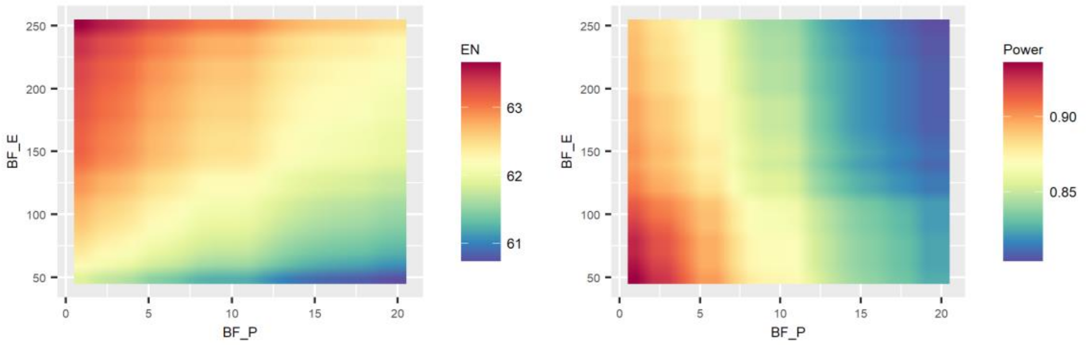

Fig. C2 Operating characteristics of the two-stage adaptive enrichment design with varying decision thresholds under scenario 2.

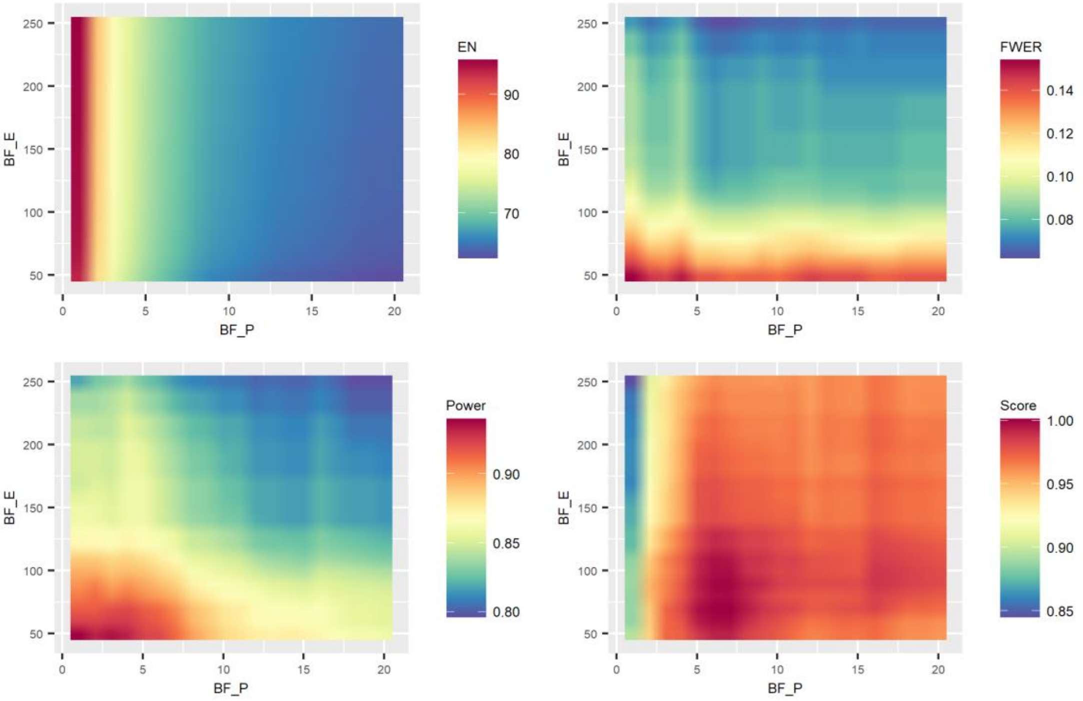

Fig. C3 Operating characteristics of the two-stage adaptive enrichment design with varying decision thresholds under scenario 3.

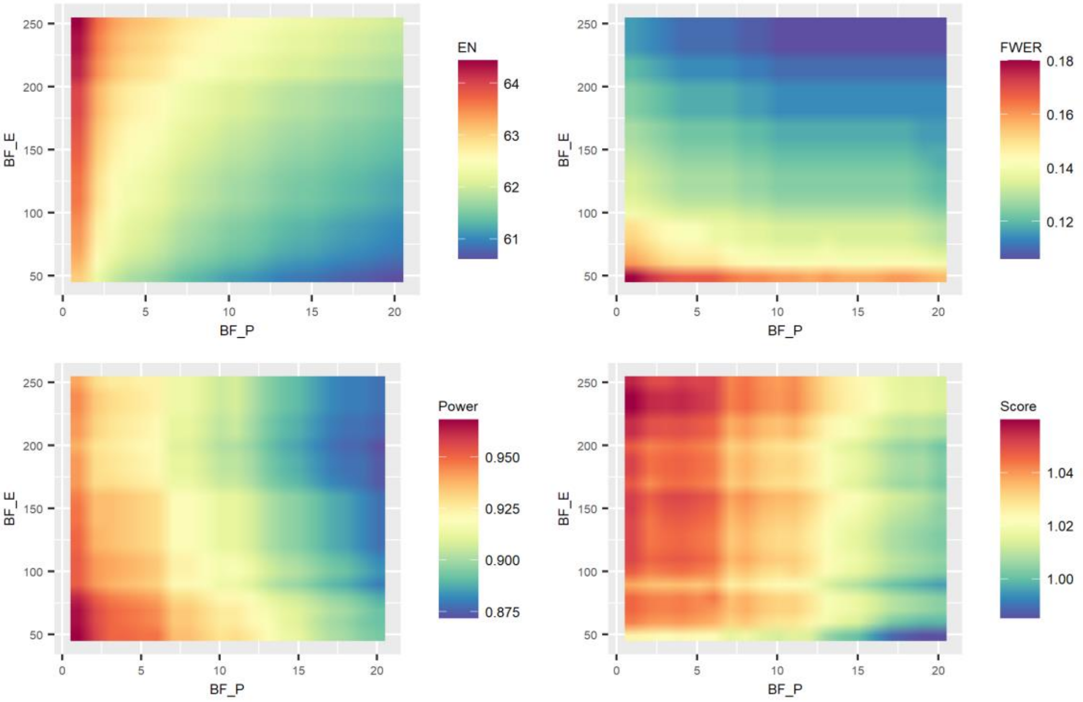

Fig. C4 Operating characteristics of the two-stage adaptive enrichment design with varying decision thresholds under scenario 4.

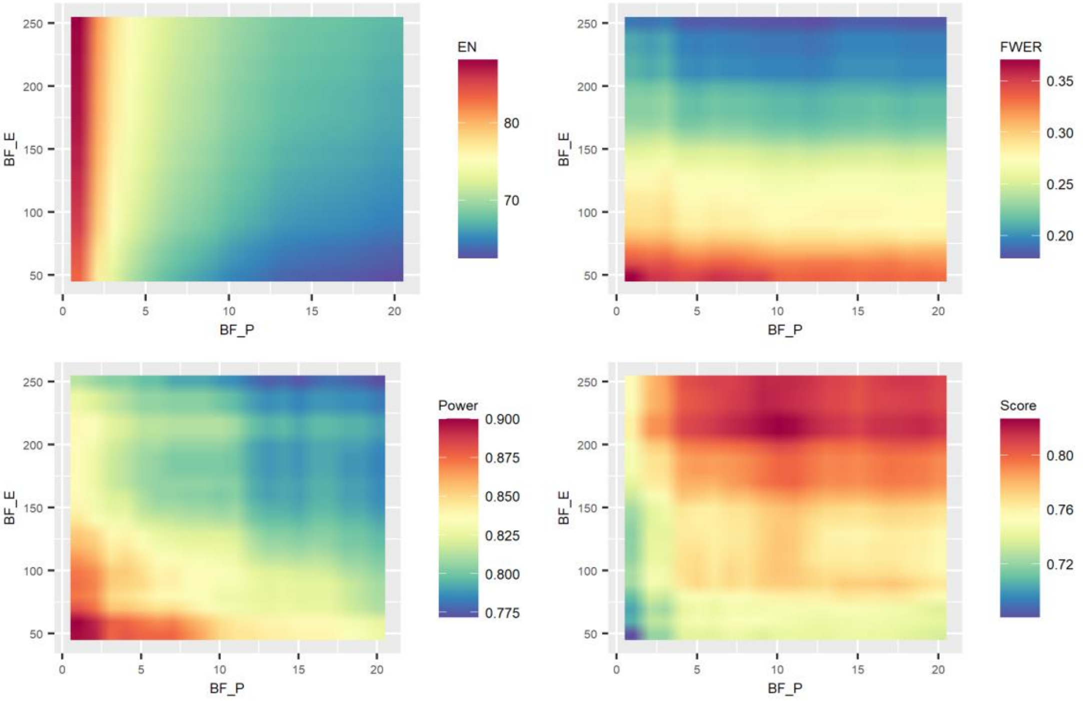

Fig. C5 Operating characteristics of the two-stage adaptive enrichment design with varying decision thresholds under scenario 5.

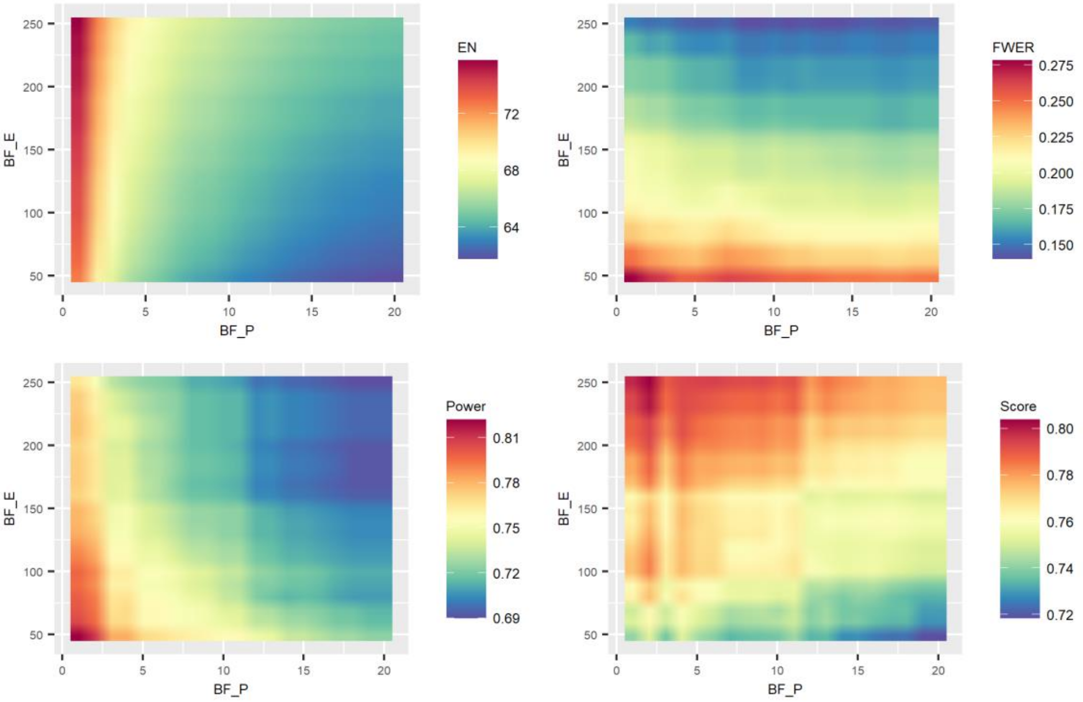

Fig. C6 Operating characteristics of the two-stage adaptive enrichment design with varying decision thresholds under scenario 6.

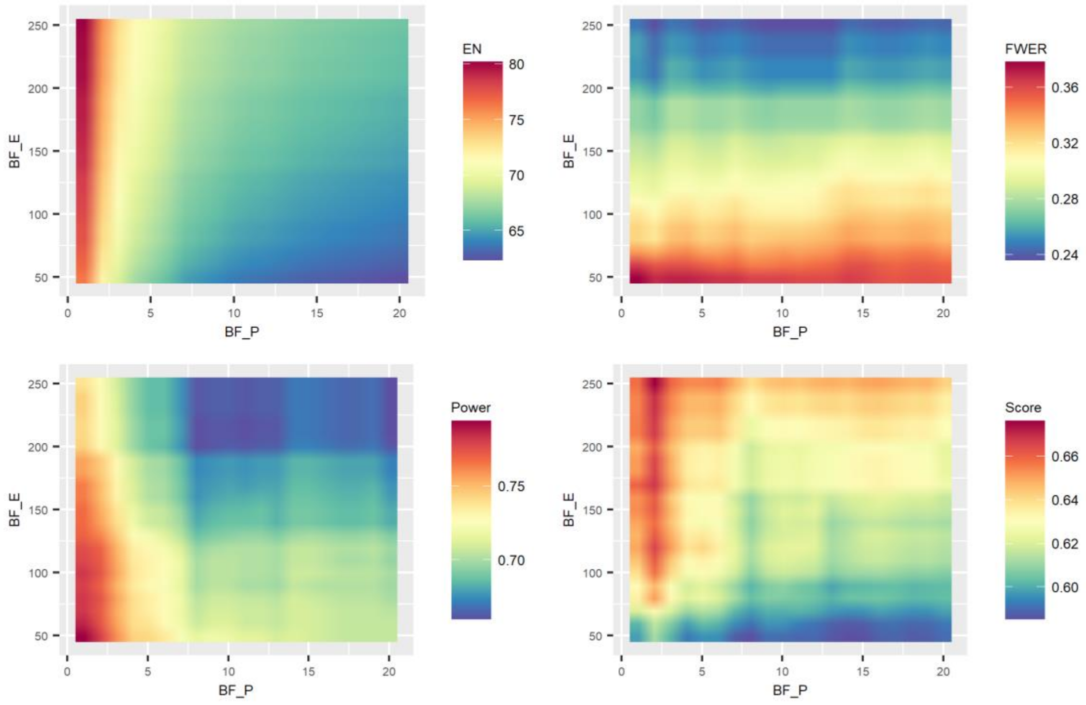

Fig. C7 Operating characteristics of the two-stage adaptive enrichment design with varying decision thresholds under scenario 7.
